# Research Progress on Genotoxicity and Molecular Mechanisms of Environmental Endocrine Disruptors

**DOI:** 10.3390/toxics14100882

**Published:** 2026-09-30

**Authors:** Junmei Xia, Jiaying Li, Yingxin Yang, Xiaoyu Lu, Bingli Lei, Tian Chen

**Affiliations:** 1 Institute of Environmental Pollution and Health, School of Environmental and Chemical Engineering, Shanghai University, Shanghai 200444, China18221160163@163.com (X.L.); 2 Department of Environmental Health, Shanghai Municipal Center for Disease Control and Prevention, Shanghai 200336, China; 3 State Environmental Protection Key Laboratory of the Assessment of Effects of Emerging Pollutants on Environmental and Human Health, Shanghai Municipal Center for Disease Control and Prevention, Shanghai 200336, China; 4 NMPA Key Laboratory for Monitoring and Evaluation of Cosmetics, Shanghai Municipal Center for Disease Control and Prevention, Shanghai 200336, China

**Keywords:** environmental endocrine disruptors, genotoxicity, toxicity mechanism, animal models, cell models

## Abstract

Genotoxicity can be transmitted across generations through germ cells, thereby substantially increasing long-term health risks. Environmental endocrine disruptors (EEDs) are ubiquitously present, resistant to degradation, and prone to biomagnification along food chains, ultimately leading to human exposure and multi-level genetic damage (from chromosomal aberrations to DNA strand breaks and base modifications). At first, this review outlines the five major sources of EEDs and summarizes current genotoxicological detection approaches, including the Ames test, comet assay, micronucleus test, γH2AX-DDR co-detection, error-corrected sequencing, and multi-omics technologies. Subsequently, based on classical animal and cellular models, we systematically evaluate the research progress and mechanistic insights into the genotoxicity of both traditional and emerging EEDs over the past fifteen years. Our review reveals that bisphenols, phthalates, polybrominated biphenyls (PBDEs), and some novel contaminants (e.g., BPA substitutes and PFAS alternatives) consistently induce DNA damage, chromosomal abnormalities, and micronucleus formation. The underlying core mechanisms converge on genotoxic insult driven by reactive oxygen species (ROS)-mediated oxidative stress, interference with DNA damage response (DDR) pathways, and epigenetic dysregulation. Notably, some novel BPA substitutes exhibit even greater toxicity than their parent compounds, and combined exposures, even at environmentally relevant low doses, can provoke significant genetic lesions and pose transgenerational risks. In light of current knowledge gaps, we propose that future research should leverage high-throughput screening, integrative multi-omics, and artificial intelligence to focus on low-dose, long-term, and co-exposure scenarios, thereby providing robust scientific evidence for genotoxicity risk assessment. This review not only supplies foundational data for evaluating the genotoxic hazards and health risks of EEDs to humans, but also delineates a forward-looking roadmap for environmental genetic toxicology research.

## 1. Introduction

With the rapid development of industry, a large number of synthetic compounds are released into the environment during their production and use, posing serious threats to both ecosystems and human health. Among them, a class of substances that can interfere with the normal functions of the endocrine system including hormone synthesis, secretion, transport, receptor recognition, and feedback regulation, are defined as environmental endocrine disruptors (EEDs) [1]. Extensive surveys have revealed the frequent detection of EEDs in environmental media such as soil, water, and sediments across various countries and regions [2]. The environmental occurrence levels of typical EEDs in surface water, wastewater and sediment are summarized in Table A1. Human exposure to these compounds is also widespread. Over the past two decades, typical EEDs including pesticides and plasticizers have been repeatedly detected in human samples such as serum, urine, placental tissue, and breast milk [3].

Given the pivotal role of the endocrine system in maintaining homeostasis and regulating reproduction, development, and behavior, EEDs can disrupt the synthesis and metabolism of endogenous hormones, either activating or suppressing endocrine system functions, subsequently resulting in neurotoxicity, immunotoxicity, and reproductive toxicity across multiple systems. Among the various toxic effects of EEDs, genotoxicity has garnered particular attention, as it is directly linked to mutagenicity, teratogenicity, and even the development of certain cancers [4]. Genotoxicity refers to the damage caused by chemical substances interacting with DNA or other cellular targets responsible for maintaining the integrity of genetic material, such as the formation of DNA adducts, strand breaks, point mutations, and changes in the structure and number of chromosomes [5], which are also regarded as direct genotoxic endpoints representing measurable alterations to genetic material itself. By contrast, oxidative stress, altered gene expression, and cell apoptosis are regarded as indirect effects, which can mediate or promote the occurrence and hazard of genotoxicity. In the absence of carcinogenicity data, genotoxicity tests are often employed to predict the potential carcinogenic risks of chemical substances [6]. Therefore, research on the genotoxicity of EEDs has always been a hot topic in the field of environmental toxicology.

For clarity, key relevant toxicological endpoints are distinguished. DNA damage refers to molecular-level lesions of DNA molecules. Genotoxicity describes the capability of a chemical to induce DNA- or genome-level damage. If unrepaired DNA damage becomes stably heritable sequence alterations, it manifests as mutagenicity. Subsequent accumulation of such mutations may eventually contribute to carcinogenicity. By contrast, oxidative stress, DNA-damage-response (DDR) activation and apoptosis represent intermediate cellular stress-response events; they do not equate to genotoxicity or DNA damage, though they can be closely linked to these adverse outcomes. Based on the above background, this review summarizes the experimental findings on the genotoxicity of EEDs over the past 15 years. It systematically categorizes the genotoxic effects induced by typical EEDs in animals or humans and delves into their underlying mechanisms of action. The aim of this review is to provide a comprehensive understanding of EED-induced genotoxicity and a new perspective and useful reference for future in-depth investigations into the genotoxicity mechanisms.

## 2. Methodology

This is a narrative review; thus, no PRISMA flow diagram is required. Databases, search terms, time frame, and study eligibility criteria are described below.

To systematically retrieve relevant literature, this study conducted a comprehensive search across the Web of Science, ScienceDirect, and China National Knowledge Infrastructure (CNKI) databases using the following keywords: “genotoxicity”, “environmental endocrine disruptors”, “chromosomal aberration”, “DNA damage”, “gene mutations”. The search time frame was set from 2011 to 2026.

The retrieved literature covered a broad range of disciplines, including environmental chemistry, toxicology, food science and technology, environmental science, genetic toxicology, environmental molecular biology, ecotoxicology, cell biology, aquatic biology, and agronomy. The initial database search yielded 335 records. After deduplication, preliminary screening based on titles and abstracts was performed, and 216 records were selected for full-text assessment.

Inclusion criteria:

- Original research articles investigating genotoxic effects, underlying molecular mechanisms, or genotoxicity-related detection methods for EEDs;
- Studies performed using in vivo animal models or in vitro cell models;
- Publications with publicly accessible full-text resources.

Exclusion criteria:

- Reviews without original experimental evidence;
- Papers that only described endocrine-disrupting phenotypes without genotoxicity-related endpoints;
- Conference abstracts, proceedings, and manuscripts without publicly available full-text.

Following full-text evaluation, a total of 111 publications met the objectives of this narrative review and were finally included for analysis.

## 3. Types of EEDs

In this review, substances are classified as environmental endocrine disruptors (EEDs) following the authoritative WHO-UNEP scientific assessment framework. Chemicals are defined as EEDs if they can interfere with hormone synthesis, secretion, transport, receptor recognition, or feedback regulation of the endocrine system. According to the results of a joint scientific assessment by the WHO and UNEP, close to 800 chemicals are known or suspected to interfere with endocrine-related molecular targets [7]. Based on their primary source pathways, they can be broadly classified into five major categories: industrial sources, agricultural sources, consumer product sources, combustion and by-product sources, and emerging environmental pollutants [3].

Industrial sources primarily encompass plastic raw materials and plasticizers, as well as flame retardants and insulating materials. Bisphenol compounds serve as the fundamental building blocks for polycarbonate plastics and epoxy resins, among which bisphenol A (BPA) is the most extensively produced and widely applied [8]. Owing to its confirmed estrogenic activity and toxicity, BPA has been progressively replaced by its structural analogs in recent years, including bisphenol F (BPF), bisphenol S (BPS), bisphenol AF (BPAF), bisphenol B (BPB), bisphenol Z (BPZ), bisphenol AP (BPAP), and others [9,10]. Phthalate esters (PAEs) represent the most commonly used plasticizers and are broadly incorporated into polyvinyl chloride (PVC) products. The major representatives include di(2-ethylhexyl) phthalate (DEHP), dibutyl phthalate (DBP), benzyl butyl phthalate (BBP), dimethyl phthalate (DMP), and diethyl phthalate (DEP) and the next-generation plasticizer diisononyl cyclohexane-1,2-dicarboxylate (DINCH) [11,12]. As for insulating and flame-retardant materials, they mainly consist of polychlorinated biphenyls (PCBs), which were historically used as insulating oils in power capacitors and transformers, alongside polybrominated biphenyls (PBDEs) [13].

Various chemical inputs used in agricultural production constitute a significant source of EEDs. These can be broadly categorized into four groups. The first one is organochlorine pesticides (OCPs) and typical representatives include dichlorodiphenyltrichloroethane (DDT), atrazine, and hexachlorocyclohexane (HCH) [14]. Owing to their lipophilicity and toxicity, most of these compounds have been banned [15]. However, they remain detectable in soil and water due to their persistence. The second one is organophosphorus pesticides that are extensively used as substitutes of OCPs and representative compounds include dimethoate, malathion, and acephate [16]. The third one is pyrethroid insecticides [17]. Although classified as highly effective and of low acute toxicity, prolonged exposure may still pose potential genotoxic risks. The last one is herbicides and major representatives include linuron, nitrofen, and prometryn. Owing to their large-scale application, they induce notable genotoxic effects on aquatic and soil organisms [18,19].

The production and use of everyday consumer items also release various EEDs into the environment. Based on their functions and chemical properties, these compounds can be grouped into the following three categories. The first one is degradation products of detergents and surfactants. The main compounds such as nonylphenol and octylphenol are environmental breakdown products of alkylphenol polyethoxylate surfactants commonly used in detergents [20]. The second one is preservatives and bactericides. These are widely added to cosmetics, skin care products, toothpastes, and food preservation materials and major examples include triclosan (TCS), parabens, and benzalkonium chloride [21]. The third one is pharmaceuticals and personal care products (PPCPs), which include natural or synthetic hormones like 17β-estradiol and 17α-methyltestosterone as well as commonly used drugs such as carbamazepine and diclofenac [22]. Carbamazepine is included here based on reported potential endocrine-perturbing properties from aquatic toxicological assays with *Daphnia magna*, while its definite endocrine-disrupting potency has not been fully established [23].

Combustion and by-product sources primarily encompass pollutants generated directly during combustion processes, as well as by-products formed through subsequent oxidation or disinfection reactions. It can be further divided into the following three subcategories. The first one is plastic incineration products. The incineration of chlorine-containing plastic materials releases persistent organic pollutants (POPs), including dioxins and furans [2]. The second one is drinking water disinfection by-products (DBPs). During chlorination, the reaction between chlorine and organic matter in water generates a variety of DBPs, such as haloacetic acids, halobenzoquinones and chloroform. These compounds commonly possess certain genotoxic properties [24]. Among them, formaldehyde has been suggested to exert potential endocrine-relevant effects based on human placental trophoblast experimental observations, yet supporting evidence remains limited [25]. The third one is tire wear oxidation products. The antioxidant N-(1,3-dimethylbutyl)-N′-phenyl-p-phenylenediamine (6PPD) used in tire rubber can be transformed by ozonation into 6PPD-quinone (6PPD-Q). This transformation product has been shown to exhibit potent genotoxicity in aquatic organisms and has thus attracted considerable research attention in recent years [26].

In addition, in recent years, a number of emerging contaminants of great concern have been identified as novel EEDs with potent endocrine-disrupting and genotoxic properties. These include per- and polyfluoroalkyl substances (PFAS), novel organophosphate flame retardants (OPFRs), as well as microplastics and nanoplastics (MPs/NPs). Notably, polymer particles of MPs/NPs themselves are not classified as endocrine-disrupting chemicals; their endocrine-disrupting and genotoxic risks mainly stem from incorporated or sorbed EED-relevant additives (e.g., bisphenols, phthalates, PBDEs), leachable constituents, and particle-provoked inflammatory responses [27]. PFAS are a class of synthetic fluorinated organic compounds, with typical representatives such as perfluorooctanoic acid (PFOA), perfluorooctane sulfonate (PFOS), and their substitutes [28]. Novel OPFRs, such as tris(1-chloro-2-propyl) phosphate (TCPP), tris(2-chloroethyl) phosphate (TCEP), and tris(1,3-dichloro-2-propyl) phosphate (TDCPP), have been increasingly employed as substitutes for traditional BPDEs in furniture, electronic products, and building materials in recent years [29]. These compounds are reported to exhibit potential endocrine-disrupting activity, though available evidence remains inconsistent across studies [30]. Recent studies have confirmed the genotoxic and potential carcinogenic risks of some of these substances, rendering them priority targets for current research in environmental genotoxicity [31,32].

## 4. Genotoxicity Testing Methods for EEDs

Genotoxicity endpoints are generally classified into three major categories: gene mutations, chromosomal and genomic aberrations, and DNA damage [33,34]. Accordingly, the testing methods adopted vary depending on the specific endpoints of interest.

Comet assay, also known as single-cell gel electrophoresis (SCGE), was originally developed by Östling and Johanson in 1984 as a neutral single-cell gel electrophoresis technique, and later refined into the widely adopted alkaline comet assay by Singh et al. in 1988 [35]. It is a rapid technique for detecting DNA damage at the single-cell level. The principle of the comet assay involves embedding cells in agarose gel, lysing the cells to remove membranes and proteins while retaining supercoiled DNA, and then subjecting the slides to alkaline or neutral conditions to unwind the DNA. Upon electrophoresis, fragmented DNA from damaged cells, due to its smaller molecular weight, migrates from the nucleus toward the anode, forming a “comet-like” tail, whereas undamaged cells, with intact high-molecular-weight DNA, remain as round fluorescent foci [36]. By measuring parameters such as tail length and tail DNA content, the extent of single- and double-strand breaks can be quantitatively assessed. More severe damage results in longer tails with higher fluorescence intensity [37]. Based on the pH of the electrophoresis buffer, the assay is divided into the alkaline comet assay and the neutral comet assay [35]. The former is more sensitive and can detect both single- and double-strand breaks as well as alkali-labile sites, and is therefore the most widely used version and the latter primarily detects double-strand breaks [36]. Owing to its sensitivity, versatility, and accuracy, it has been widely applied in genotoxicity testing, human biomonitoring, molecular epidemiology, ecotoxicological studies, and fundamental research on DNA damage and repair [38,39]. When testing endocrine-disrupting pollutants, this assay offers high sensitivity toward single- and double-strand DNA breaks in fish erythrocytes and cultured cells.

The Ames test is known as the bacterial reverse mutation assay) was established in 1975 and remains a classic in vitro method for detecting mutagenicity using microbial gene mutation [40]. Its core principle relies on histidine-auxotrophic strains of *Salmonella typhimurium*, which cannot grow on media lacking histidine. When a test chemical is mutagenic, it induces reverse mutations in the defective strains, restoring them to the wild-type (histidine-prototrophic) phenotype, thereby enabling colony formation on selective medium. The number of colonies directly reflects the mutagenic potential of the test substance. To mimic mammalian metabolic activation, rat liver microsomal fraction (S9) is typically added to detect promutagens that require metabolic conversion [41]. The Ames test is rapid, simple, and widely employed for initial genotoxicity screening of pesticides, food additives, and environmental pollutants, and is particularly suitable for evaluating the combined mutagenic effects of complex mixtures [6]. In EED-related toxicological research, this assay is widely applied for high-throughput preliminary mutagenicity screening of candidate endocrine-disrupting chemicals; nevertheless, it cannot recapitulate eukaryotic cellular or in vivo physiological conditions.

Error-corrected sequencing is an innovative technology based on next-generation sequencing (NGS) [42]. Its core principle involves attaching unique molecular identifiers to each original DNA molecule, allowing the identification and correction of random errors introduced during PCR amplification or sequencing by comparing multiple reads derived from the same original molecule. This enables ultra-sensitive detection of low-frequency mutations. In contrast to the traditional Ames test, which relies on phenotypic reversion in auxotrophic bacteria and primarily detects point mutations with limited sensitivity, error-corrected sequencing can directly identify various types of low-frequency mutations across the whole genome. Therefore, it is especially suitable for precise detection of subtle mutations induced by low-dose EED exposure, combined pollution, and transgenerational effects, offering higher resolution and richer information than the Ames test for environmental genotoxicity assessment. This cutting-edge sequencing tool enables detection of low-frequency somatic mutations induced by EED exposure that escape conventional genotoxicity assays. Even so, high experimental cost and complicated bioinformatic workflows limit its application for large-sample-size preliminary screening.

The micronucleus test, developed independently by Heddle and Schmid in the 1970s, is a classic tool for genotoxicity screening of chemicals and particularly effective for detecting clastogens and aneugens [43]. The in vitro micronucleus test is widely used to assess chromosome damage caused by chemicals. The underlying principle is that during mitosis, if a test substance causes chromosome breakage or spindle dysfunction, chromosome fragments or entire lagging chromosomes fail to be incorporated into daughter nuclei at telophase and instead form one or more small, secondary nuclei in the cytoplasm, termed micronuclei [43]. The micronucleus assay is frequently adopted to evaluate chromosome damage induced by EEDs such as bisphenols and phthalates in cultured cells and experimental fish; nevertheless, it cannot directly identify primary DNA-strand-break lesions.

Combined γH2AX and DDR analysis enables simultaneous assessment of DNA double-strand break (DSB) signaling and downstream repair pathway activation [44]. The principle is that upon DSB formation, histone H2AX is rapidly phosphorylated at Ser-139 (forming γH2AX) at the break sites, serving as a sensitive marker of DSBs. Concurrently, a series of DDR proteins (e.g., ATM, 53BP1, BRCA1) are recruited to the damage sites, initiating downstream repair cascades [45]. Simultaneously detecting γH2AX foci and the activation/recruitment of DDR proteins can not only identify the presence and quantity of DSBs but also evaluate the activation status of repair pathways, thus distinguishing between “simple damage” and “damage-initiated repair” [31]. Compared with measuring γH2AX alone, which only reflects DNA damage occurrence without indicating subsequent repair capacity, the combined approach provides a more comprehensive evaluation of the impact of compounds on the entire DNA damage-repair process. Consequently, this combined strategy has been extensively applied in mechanistic studies of EED-induced genotoxicity and is regarded as one of the gold-standard methods for assessing DNA damage and genotoxicity risk [14,46]. For cellular EED toxicology work, this integrated strategy goes beyond simple DNA double-strand break marking and characterizes the full activation status of DNA-damage-response signalling networks. Still, it is largely restricted to in vitro cell systems and difficult to implement directly within intact animal tissues.

With the continuous advancement and widespread adoption of high-throughput sequencing and high-resolution mass spectrometry technologies, multi-omics integration analysis techniques rapidly matured and were widely adopted after 2015 [47]. These systematic analytical approaches utilize platforms such as whole-genome sequencing, transcriptome sequencing, proteome sequencing, and liquid chromatography-tandem mass spectrometry (LC-MS/MS) to comprehensively capture changes in the abundance, composition, and modifications of DNA, RNA, proteins, and metabolites across multiple molecular layers. Multi-omics strategies can efficiently screen for specific biomarkers of genotoxicity and uncover cryptic genetic damage and perturbed pathways that traditional endpoint assays (e.g., gene mutation or chromosomal aberration tests) may miss [48]. These multi-layer profiling techniques help reveal complex molecular signatures triggered by EED insult. A major caveat is the difficulty in discriminating bona fide direct genotoxic outcomes from secondary molecular changes driven by general cellular stress.

In short, these genotoxic assays have their respective strengths and drawbacks for EED assessment. Ames test is suitable for preliminary mutagenicity screening. Micronucleus assays reflect chromosome-level damage. The comet assay is sensitive to DNA-strand breaks but requires strict standardization. γ-H2AX-based tools mark double-strand-break signals at the cellular level. Advanced sequencing and multi-omics workflows provide high-resolution molecular profiling, yet they are often cost-intensive or cannot readily separate direct genotoxicity from indirect stress-related responses. Combining several complementary assays is preferred to obtain robust genotoxicity evidence for EEDs. The main features, endpoints, principles and limitations of these genotoxicity detection technologies are summarized in Table 1.

Nevertheless, despite the wide array of available genotoxicity assays, each test targets a specific endpoint and operates on distinct mechanistic principles, failing to cover the full spectrum of DNA damage. This limitation may lead to inconsistencies among test results and even the oversight of potential genotoxic risks of certain EEDs [49]. Therefore, there is an urgent need to develop next-generation genotoxicity testing and chemical screening systems that are more economical, rapid, yet simultaneously high-throughput, information-rich, and reliable, in order to fill current methodological gaps [50].

**Table 1:** Detection technologies for genotoxicity evaluation of EEDs.

Experimental Methods	Genotoxic Endpoints	Principle	Limitations	References
Ames test	Gene mutation	This assay employs histidine auxotrophic mutant strains of *Salmonella typhimurium*. If the test substance is mutagenic, it can induce reverse mutations in the mutant strains to restore their ability to synthesize histidine, and mutagenicity is assessed by counting the number of colonies formed on the culture medium.	Prokaryotic bacterial system differs from mammalian eukaryotic cells; only detects gene-level point/frameshift mutations, cannot assess chromosomal damage. Requires testing both with- and without-S9 to capture direct and indirect mutagens; risk of false-positive or false-negative outcomes for complex mixtures.	[51]
TK gene mutation assay	Gene mutation	Uses heterozygous TK^+^/^−^ mammalian cell lines. After chemical exposure (with-/without S-9 metabolic activation), a phenotypic-expression period is allowed. TK-deficient mutants gain resistance to trifluorothymidine (TFT). Mutant-colony frequency is counted to detect point mutations, deletions and chromosomal rearrangements.	In vitro system; exogenous S-9 cannot fully recapitulate in vivo metabolism. Cannot reliably detect pure aneugenic agents under standard cytotoxicity criteria. Only monitors mutations at the single-TK locus; results are confounded by cytotoxicity, pH-osmolality artifacts and culture conditions.	[52]
Transgenic mouse gene mutation assay	Gene mutation	Uses transgenic rodents with bacteriophage-derived reporter transgenes. After in vivo exposure and mutation-manifestation period, DNA from target tissues is rescued in bacteria; mutant-plaque frequency quantifies in vivo gene-mutation events in somatic and male germ cells.	In vivo animal assay with high cost and ethical constraints. Only detects mutations in exogenous reporter transgenes, not endogenous-gene or large-scale chromosomal damage. Results depend on proper mutation-manifestation time and vary across tissues; female germ-cell mutation assessment is limited.	[53]
Error-corrected sequencing	Gene mutation (low-frequency mutation)	Attaches unique-molecular-identifiers to original DNA molecules. Consensus reads eliminate PCR-sequencing artifacts, enabling ultrasensitive detection of genome-wide low-frequency point mutations.	High sequencing-computational cost; lacks unified standardized workflows. Poor performance for large structural/chromosomal rearrangements; prone to library-preparation-derived technical artefacts.	[54]
Micronucleus assay	Chromosome damage	Detects chromosomal damage; acentric fragments or lagging whole chromosomes fail to incorporate into daughter-cell nuclei during telophase and form cytoplasmic micronuclei. Cytochalasin-B blocks cytokinesis to score only binucleated cells completing one nuclear division.	Cannot distinguish clastogenic fragments from aneugenic lagging chromosomes without centromere-/kinetochore-specific staining. Confounded by cytotoxicity-mediated cell-cycle inhibition; only works in dividing cells; manual scoring is labour-intensive and subject to scorer bias.	[43]
Comet assay	DNA damage (single-/double-strand breaks, SSBs/DSBs)	Cells embedded in agarose are lysed to retain nucleoids with supercoiled DNA. Under high-pH electrophoresis, broken-DNA loops migrate to form comet tails; tail-to-head fluorescence ratio quantifies DNA-strand breaks. Lesion-specific endonucleases can detect oxidized-base lesions.	High inter-experimental variability; signal saturation at heavy DNA damage. Cannot distinguish lesion types without endonucleases; “hedgehog comet” artifacts interfere with scoring; does not detect gene or chromosome mutations.	[35]
γ-H2AX foci immunofluorescence assay	DNA damage (double-strand breaks, DSBs)	Detects DNA double-strand breaks (DSBs). Upon DSB formation, histone H2AX is phosphorylated to γ-H2AX at break sites, forming discrete nuclear foci visualized by immunofluorescence; foci number per nucleus quantifies DSB burden.	Not exclusively DSB-specific; foci can arise from replication stress or apoptotic DNA fragmentation. No unified scoring standard; manual counting suffers operator bias; cannot detect single-strand breaks or base lesions directly.	[55]
Combined detection of γ-H2AX and DNA damage response	DNA-damage-triggered signalling and double-strand breaks	Multiplex flow-cytometric detection combining γ-H2AX (DSB marker) with DDR-related biomarkers including p53 and phospho-histone H3. Multiparameter signals at multiple time-points are integrated to discriminate clastogenic, aneugenic and non-genotoxic agents, assisting genotoxic mode-of-action interpretation.	Restricted largely to TK6 cell system; high cytotoxicity can produce confounding signals. Dependent on specialized flow-cytometry hardware and machine-learning classification trained on reference chemicals; lacks globally harmonized protocols; cannot directly score base lesions, gene or chromosomal mutations.	[56]
Detection of oxidative stress biomarkers	Redox-mediated pre-genotoxic stress	Detects cellular reactive-oxygen/nitrogen species, antioxidant-response or oxidized-DNA lesions (e.g., 8-oxodG) to assess redox imbalance linked to potential secondary genotoxic effects.	Oxidative-stress signals do not equate to genotoxic risk; markers are transient and affected by endogenous baseline and sampling conditions; cannot directly score gene- or chromosome-level damage.	[57]
Toxicogenomic (transcriptomic) analytical techniques	Systems-level molecular-pathway perturbations related to genotoxic stress	Profiling transcriptome-wide gene expression alterations; utilizes transcriptomic biomarker signatures, connectivity mapping and hierarchical clustering to detect disturbed cellular signalling pathways and support genotoxic mode-of-action interpretation.	Assay performance is cell-model-dependent and sensitive to differential-gene-calling statistical thresholds. It cannot reliably classify all genotoxic agents when used alone and is unable to directly measure gene- or chromosome-level mutagenic phenotypic endpoints.	[58]
Epigenetic damage detection assay	Chromatin-level epigenetic perturbations without primary DNA-sequence alteration	Detects chemically induced epigenetic modification changes including DNA methylation, histone modifications and non-coding RNA profiles; captures chromatin-state perturbations to support mechanistic interpretation of carcinogenic modes-of-action.	Epigenetic marks are dynamic and reversible; it is difficult to discriminate direct toxic effects from secondary stress-driven alterations. Lacks globally standardized protocols and regulatory acceptance as standalone assay; cannot directly score gene- or chromosome-level mutagenic endpoints.	[59]

## 5. In Vivo and In Vitro Genotoxicity Studies of EEDs

### 5.1. In Vivo Studies Using Animal Models

**Figure 1:**
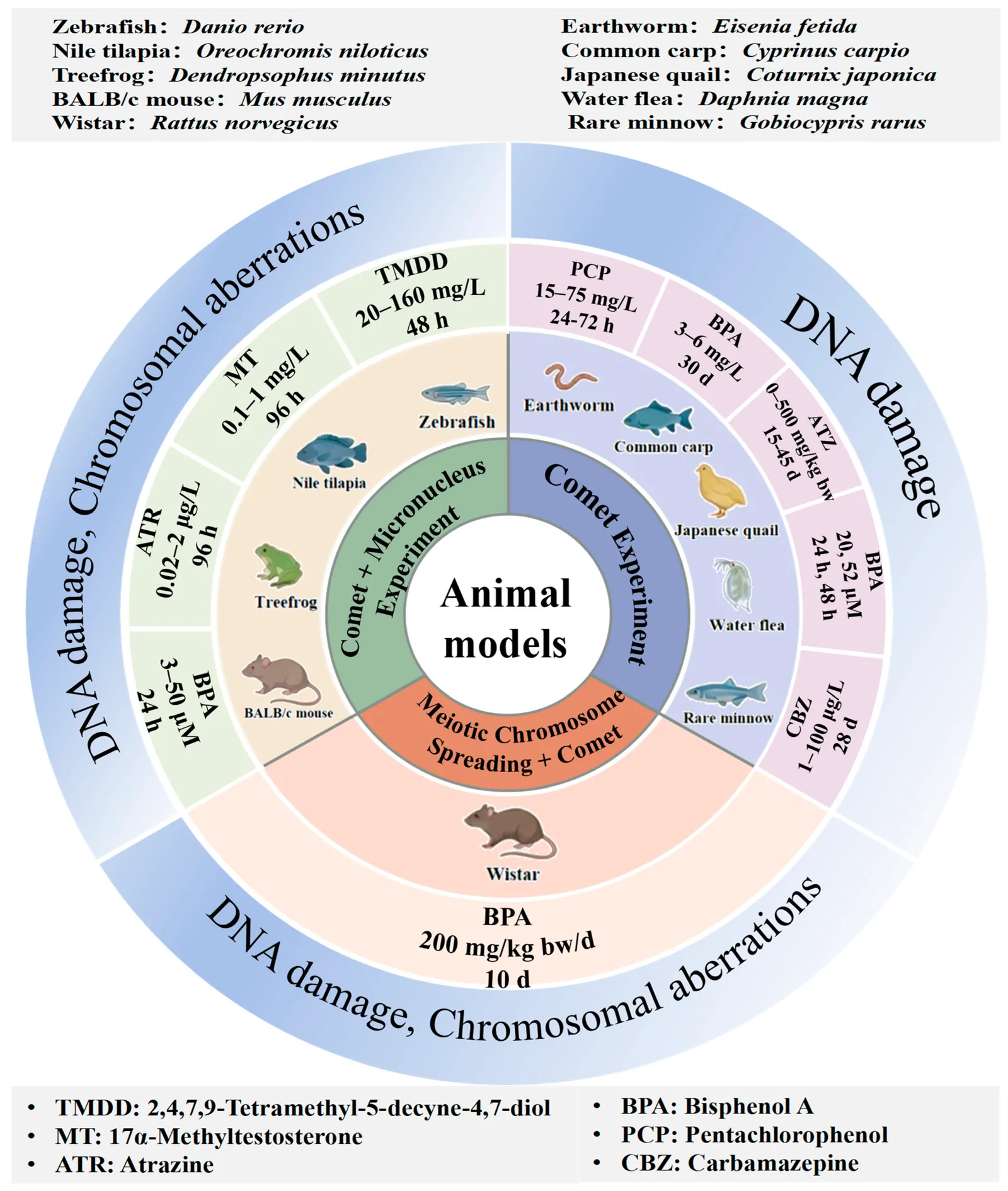
Overview of genotoxicity evaluation of EEDs using in vivo animal models.

EEDs can directly induce DNA damage, elicit genotoxic effects, and even increase carcinogenic risk following long-term exposure [33]. Figure 1 summarizes recent genotoxicity studies of EEDs across various organisms. The specific information about exposure concentrations, exposure conditions, effect endpoints, experimental methods, and key findings can be found in Table S1.

Rats and mice are the most commonly used mammalian models in toxicology [47,60]. Prethroid insecticide imiprothrin exposure can induce DNA damage and chromosomal aberration in rats using the comet assay and chromosomal aberration test [17]. Liao et al. [61] employed the comet assay to evaluate DNA damage in normal and OVA-immunized mice exposed to toluene, and found that toluene exposure induced oxidative DNA damage and elicited genotoxicity in several brain regions. Notably, the hippocampus of control mice exhibited higher susceptibility to toluene-induced genotoxicity compared to OVA-immunized mice, suggesting that immune status may influence the susceptibility to chemical-induced neurogenotoxic effects.

As an ecologically relevant macroinvertebrate that is abundant in aquatic ecosystems, the midge Chironomus has been adopted as a standard test organism for evaluating toxicity of EEDs in international standardized protocols. Martínez-Paz et al. [62] used the comet assay to evaluate the genotoxicity of five EEDs including BPA, nonylphenol (NP), pentachlorophenol (PCP), tributyltin (TBT), and triclosan (TCS) on aquatic larvae of *Chironomus riparius*, and found that all tested compounds induced DNA damage. Notably, in most treatment groups, prolonged exposure did not correspondingly increase the extent of DNA damage, suggesting that the genotoxicity of these compounds in Chironomus is not simply time-dependent. Aquilino et al. [5] assessed the DNA damage potential of fungicide vinclozolin (Vz) on Chironomus larvae with the comet assay and measured transcriptional changes in DNA repair-related genes (ATM, NLK, XRCC1) and the apoptosis-related gene (DECAY) and confirmed the potential genotoxicity of Vz to freshwater invertebrates.

Fish such as zebrafish and rainbow trout are traditional vertebrate animal models. Zebrafish share high genomic similarity with humans, rendering them ideal for genotoxicity assessment [63]. Du et al. [64] employed miRNA sequencing, dual-fluorescence protein assays, and dual-luciferase reporter assays to demonstrate that exposure to environmentally relevant low-dose gradients of DEHP significantly reduced zebrafish embryo survival, accompanied by downregulation of miR-146a and upregulation of TRAF6, indicating that DEHP exerts genotoxic effects during early embryonic development, potentially through modulation of inflammation-related signaling pathways. In adult fish models, Rivero-Wendt et al. [65] evaluated the genotoxicity of the synthetic hormone 17α-methyltestosterone (MT) in adult Nile tilapia (*Oreochromis niloticus*) and the Brazilian native non-target fish (*Astyanax bimaculatus*) using the micronucleus test and comet assay. *Oreochromis niloticus* is the target aquaculture species for MT-induced sex reversal, while *Astyanax bimaculatus* serves as a local bioindicator to assess ecological risks posed by hatchery-released contaminants to natural freshwater ecosystems. They found that no significant differences in micronucleus frequency or DNA damage were observed between the two species, while MT significantly increased erythrocyte nuclear abnormalities in the non-target fish (*Astyanax bimaculatus*). Brown trout (*Salmo trutta*) represents a native salmonid bioindicator species widely used for aquatic endocrine-disruptor risk assessment; its nucleated peripheral erythrocytes are well-suited for micronucleus-based genotoxicity monitoring, and chronic-release implant exposure protocols are well-established in this species to mimic long-term environmental exposure. Frenzilli et al. [9] employed this species as a model and combined the micronucleus test with liquid chromatography-electrospray tandem mass spectrometry to confirm that both BPA and its substitute BPS induce endocrine disruption and genotoxic effects, and demonstrated that BPA and BPS trigger different DNA damage patterns, suggesting distinct mechanisms of action. Collectively, these studies indicate that different fish models offer unique advantages in evaluating EED genotoxicity, and that different species, exposure concentrations, and detection endpoints can all influence the assessment of genotoxicity.

The male Japanese quail (*Coturnix japonica*) has high sensitivity to endocrine disruptors and is a valuable avian model in environmental toxicology [66]. It has been reported that dibutyl phthalate (DBP) dose-dependently induces typical apoptotic features in spermatogenic cells, including nuclear membrane rupture, chromatin condensation, and apoptotic body formation, confirming the genotoxic effects of DBP on avian germ cells [67].

Amphibian models also offer unique advantages in genotoxicity assessment. Tadpoles, as the larval stage of amphibians, have short growth cycles, obvious morphological changes, and are easy to observe, making them ideal subjects for studying the effects of environmental pollutants on early development. Scaia et al. [68] used bullfrog (*Lithobates catesbeianus*) tadpoles as a model and employed the micronucleus test, along with binucleated, anucleated, and nuclear bud indicators, to systematically evaluate the genotoxic effects of NP. They found that NP not only induced genetic damage in tadpoles but also caused significant morphological alterations by interfering with oogenesis, inducing nuclear abnormalities, and changing body color.

In addition to aquatic and mammalian models, earthworms (e.g., *Eisenia fetida* and *Pheretima guillemi*) have emerged as valuable model organisms in terrestrial ecotoxicology for evaluating the genotoxicity of EEDs and emerging pollutants in recent years. Chen et al. [69] integrated transcriptomic and metabolomic analyses and found that soil antimony (Sb) exposure significantly disturbed the endocrine system, immune system, and oxidative phosphorylation pathways in earthworms, confirming its multi-target genotoxicity potential. For nanoplastics, although direct genotoxic endpoints were not measured in the available assays, Zhou et al. [70] found that nanoplastics accumulated in earthworms and induced oxidative stress, leading to immune coelomocyte death; these observed toxic effects may arise from particle-mediated physical impacts as well as associated chemical contaminants. These earthworm-based studies have not only filled knowledge gaps regarding the genotoxic effects of EEDs on terrestrial invertebrates but also provided critical data for assessing risks associated with soil combined pollution.

Taken together, in vivo genotoxicity assessments of EEDs rely on a diverse suite of animal models with complementary strengths and inherent limitations. Rodent models (mouse and rat) possess mammalian physiological features relevant to human health outcomes and are well-suited for investigating reproductive and somatic genotoxicity, although they are relatively costly and less applicable for field-relevant ecological monitoring. Fish-based assays constitute robust systems for aquatic ecotoxicological risk assessment, supporting investigations ranging from developmental toxicity to implant-mediated chronic-exposure experiments.

Avian models such as *Japanese quail* are particularly valuable for evaluating germ-cell-related genotoxic outcomes, as demonstrated by documented apoptotic damage to spermatogenic cells following phthalate exposure in this species. Amphibian tadpole models are highly responsive to early-life-stage genotoxic insults from EEDs, as illustrated by nuclear and chromosomal abnormalities observed in tadpole assays, making them suitable for amphibian-focused ecotoxicological risk assessment. Terrestrial earthworm models can readily detect soil-pollutant-triggered genotoxicity in coelomocyte immune cells; nevertheless, these invertebrates lack the sophisticated endocrine and gene-regulatory networks present in mammals.

No single animal model can perfectly recapitulate all aspects of human and environmental risk; therefore, combining evidence from multiple model organisms helps obtain a more comprehensive understanding of EED-associated genotoxic hazards.

### 5.2. In Vitro Studies Using Human Cell Models

**Figure 2:**
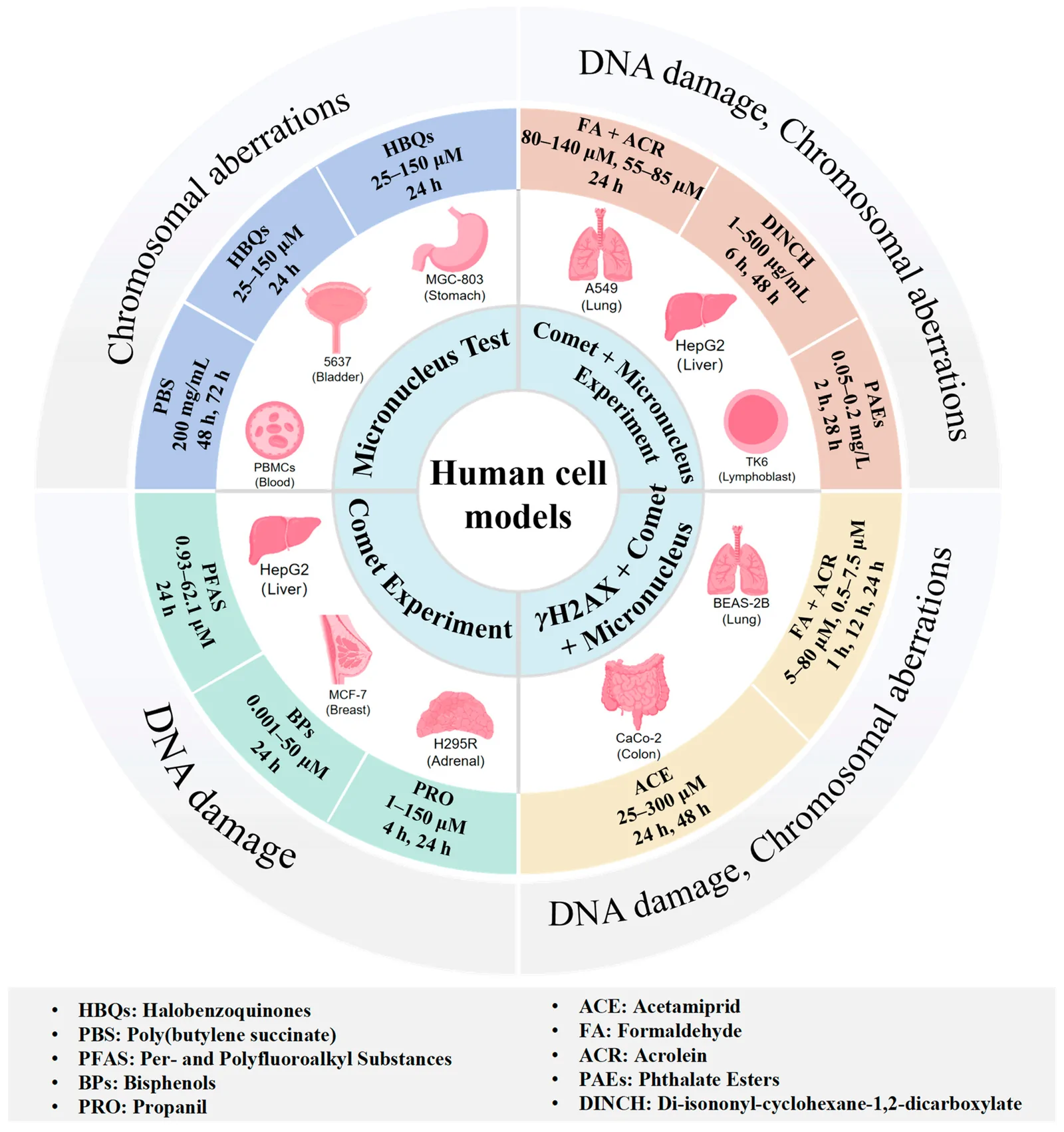
Overview of genotoxicity evaluation of EEDs using human in vitro cell models.

Animal experiments can provide in vivo results for chemical genotoxicity assessment. However, they remain costly, time-consuming, and often fail to comply with the “3R” principles. More critically, results obtained from animal tests may not accurately extrapolate to humans due to interspecies differences. In contrast, in vitro toxicity assays based on human cells are more directly relevant to human health and effectively circumvent the uncertainty of interspecies extrapolation [71]. Therefore, the establishment and optimization of human-derived cell models have become a major method in genotoxicity testing. Figure 2 summarizes recent genotoxicity studies of EEDs across various human cells and the specific information can be found in Table S2.

To date, various human cell lines have been developed for genotoxicity evaluation of EEDs. The human hepatoma cell line HepG2 possesses both hepatocyte functions and cancer cell features, making it widely used in genotoxicity experiments [13,49]. Schmidt et al. [72] employed the Ames test and comet assay to evaluate the mutagenicity and genotoxicity of eight BPA analogs (BPF, BPAF, BPZ, BPS, DMBPA, DMBPS, BP-1, and BP-2) on HepG2 cells at non-cytotoxic concentrations (0.1–10 μmol/L). The results showed that only BPA and BPS induced significant DNA damage after 24 h of exposure, whereas DMBPS, BP-1, and BP-2 caused only transient increases in DNA strand breaks after 4 h, and BPF, BPAF, BPZ, and DMBPA showed no obvious effects. While Kose et al. [10] used a modified comet assay in human normal prostate epithelial RWPE-1 cells and found that BPF and BPS could induce high levels of DNA damage at non-cytotoxic concentrations and simultaneously inhibit DNA repair pathways, indicating that different cell types exhibit differential sensitivity to bisphenol analogs.

The MCF-7 breast cancer cell line, due to its estrogen responsiveness, is a common in vitro model for endocrine disruption studies. The insecticide flumethrin induced genotoxicity in MCF-7 cells even at a low concentration of 0.1 μmol/L, as shown by micronucleus and comet assays [73]. The TK6 human lymphoblastoid cell line, which grows in suspension, is easy to culture and exhibits good sensitivity and specificity; it is frequently used in genotoxicity screening. Al-Saleh et al. [74] used the comet and micronucleus assays to evaluate the genotoxic potential of 42 perfumes in TK6 cells, finding that phthalate esters (PAEs) in perfumes significantly contribute to DNA damage. Luo [75] used human bone marrow mesenchymal stem cells (HBMSCs) as a model to study the genotoxicity of three neonicotinoid insecticides such as imidacloprid (IMI), nitenpyram (NIT), and acetamiprid (ACE) and found that all three induced DNA damage at concentrations that did not affect cell viability.

Furthermore, primary human cells offer unique advantages in reflecting real human responses [76,77]. Sicińska et al. [78] used human peripheral blood mononuclear cells (PBMCs) to investigate the epigenetic toxicity of phthalates (DBP, BBP) and their metabolites (MBP, MBzP). They found that BBP, MBP and MBzP significantly decreased global DNA methylation levels, induced hypermethylation of the promoter regions of tumor suppressor genes TP53 and P16 with consequent downregulation of their expression, and caused hypomethylation of the promoters of proto-oncogenes CCND1 and BCL2 with consequent upregulation.

For respiratory exposure pollutants, the human lung adenocarcinoma A549 cell line and normal bronchial epithelial BEAS-2B cells have become core models [79]. Multiple studies have shown that emerging EEDs such as chlorinated by-products of UV filters [80], tire oxidation product 6PPD-Q [81], and haloacetic acids [82] can induce oxidative DNA damage and micronucleus formation via ROS elevation, exhibiting genotoxicity.

### 5.3. In Vitro Studies Using Animal-Derived Cell Models

The use of animal-derived cells for in vitro genotoxicity studies represents another important approach due to their well-defined genetic backgrounds, stable proliferation, and sensitivity to oxidative stress and genetic damage. In addition, they can provide cellular and molecular mechanistic insights to complement in vivo findings. Currently, the animal cell models commonly employed in genotoxicity studies of EEDs are primarily derived from fish and mammals.

The ZFL (ZF-L) cell line, derived from zebrafish liver, is a classic in vitro model for studying hepatic metabolism, xenobiotic biotransformation, endocrine disruption, and genotoxicity in fish [83]. Studies have demonstrated that titanium dioxide nanoparticles (TiO_2_ NPs) can induce excessive ROS production in ZFL cells, leading to DNA strand breaks, chromosomal damage, and micronuclei formation [84]. In addition, primary thymic cells from the European seabass (Dicentrarchus labrax) and erythrocytes from *Oreochromis niloticus* serve as a fish-derived immune-related in vitro model that is highly sensitive to environmentally relevant concentrations of E2 and 17α-ethinylestradiol (EE2) [85,86]. Following exposure, cell proliferation is markedly inhibited, the mTOR energy checkpoint pathway is downregulated, and cell cycle progression and genomic stability are disrupted, ultimately leading to T-cell developmental arrest and immune-related genetic damage.

Mammalian cell models are also recognized as a standard in vitro system for genotoxicity assessment. Xin et al. [87] used Chinese hamster ovary (CHO) cells to systematically evaluate the cytotoxicity, genotoxicity, and mutagenicity of BPA. Zhu et al. [88] employed Mouse embryonic fibroblast (MEF) cells to investigate the combined genotoxic effects of hexavalent chromium (Cr(VI)) and 1800 MHz radiofrequency electromagnetic radiation and found a synergistic induction of DNA damage.

Compared with the above-mentioned commercially available mammalian and fish cell lines, cell models derived from amphibians and terrestrial invertebrates remain relatively limited in EED genotoxicity studies. For amphibians, embryonic cells and spermatogenic cells of *Xenopus laevis* are classic in vitro models for studying developmental and reproductive toxicity in amphibians [89,90]. For terrestrial invertebrates, earthworm coelomocytes are currently the most frequently used cell model for evaluating the genotoxicity of soil-associated EEDs using comet and micronucleus assays [91].

## 6. Genotoxicity Mechanisms

**Figure 3:**
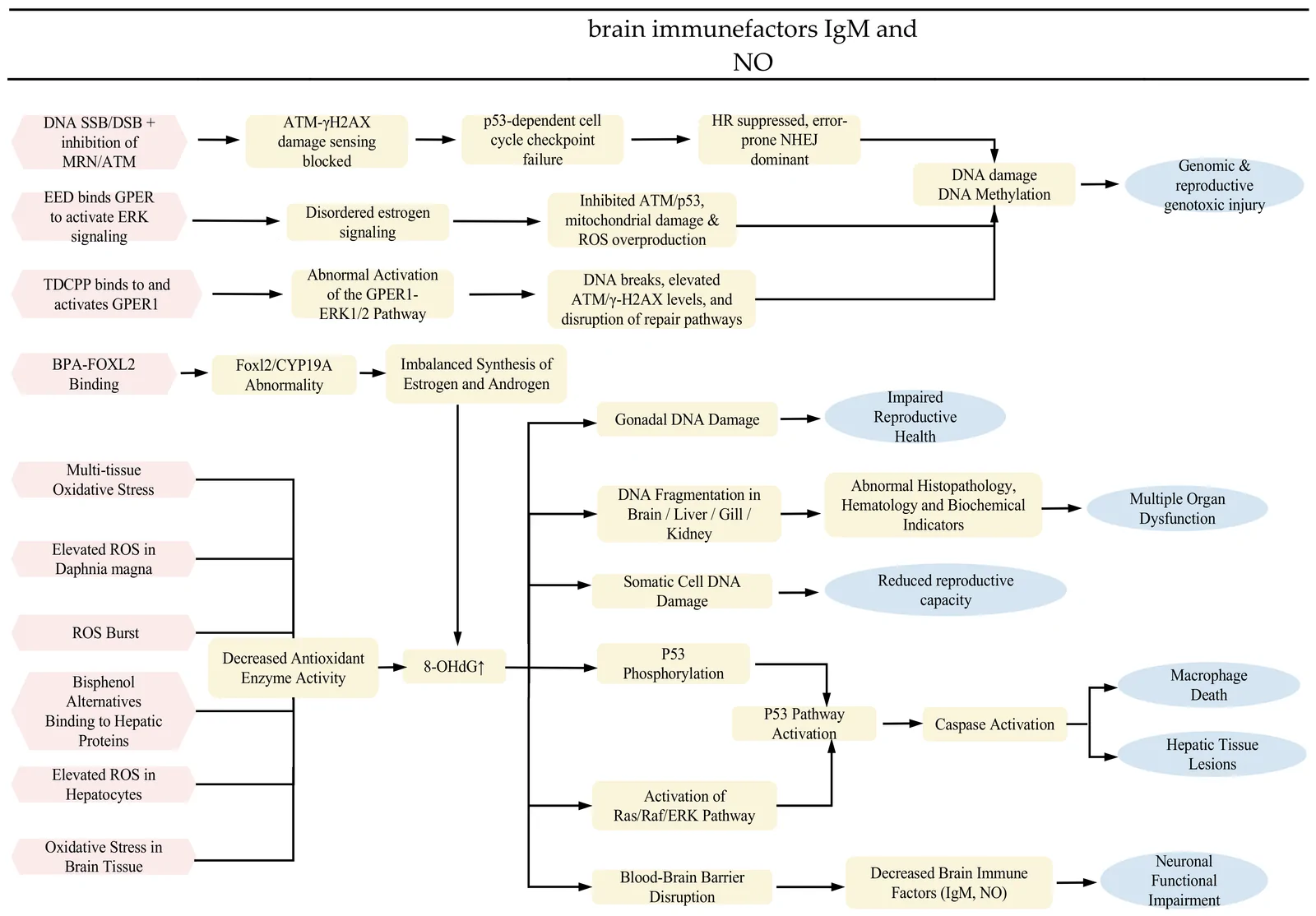
Proposed adverse outcome pathway diagram for EEDs-induced genotoxicity. Arrows denote causal sequential flow of molecular events; the upward arrow beside 8–OHdG indicates elevated levels of this oxidative DNA–damage biomarker.

To more clearly investigate the mechanisms of genotoxicity induced by EEDs, we summarized the molecular initiating events (MIEs), key events (KEs), and adverse outcomes (AOs) of EEDs-triggered genotoxicity based on published literature, as shown in Table 2. Furthermore, integrating the experimental evidence summarized in Table 2, we proposed a putative adverse outcome pathway (AOP) framework for EEDs-induced genotoxicity. As shown in Figure 3, EEDs can induce genotoxicity via multiple pathways and the core mechanisms can be summarized as ROS-mediated direct DNA damage, interference with DDR pathways, and disruption of epigenetic regulation. In addition, these pathways interact with each other and may amplify progressively, ultimately leading to accumulation of DNA damage, chromosomal aberrations, and genomic instability. Therefore, synergistic effects of combined exposure may also be an important characteristic of genotoxicity. However, this conceptual framework has not undergone formal weight-of-evidence evaluation. Further systematic experimental validation is still required to confirm the causal linkages among MIEs, KEs, and AOs.

### 6.1. ROS-Mediated Direct DNA Damage

Oxidative stress is the core upstream mechanism by which EEDs trigger direct DNA damage [98]. EEDs can disrupt mitochondrial respiratory chain function and inhibit antioxidant enzyme systems, leading to excessive accumulation of intracellular ROS and a state of oxidative stress [99]. Excessive ROS directly attack DNA molecules, inducing various forms of oxidative DNA damage, including base oxidation, single-strand breaks, and double-strand breaks, which constitute the initiating events of genotoxicity [70].

Numerous studies have confirmed that many typical EEDs initiate genotoxic effects via this pathway [100]. For example, exposure of zebrafish embryos to various exogenous aquatic environmental pollutants can lead to mitochondrial dysfunction and oxidative DNA damage, and pretreatment with the ROS scavenger N-acetylcysteine significantly alleviates the damage, confirming the critical mediating role of ROS [101,102,103]. Organophosphate flame retardants such as TCEP, TCPP, and TDCPP also increase ROS levels in human hepatoma HepG2 cells, accompanied by evident DNA strand breaks [29]. Phthalates such as DBP and DEHP can induce a significant accumulation of ROS in germ cells, which in turn leads to oxidation of sperm nucleobases and DNA strand breaks, thereby compromising sperm genomic stability [11]. Mohafrash et al. [17] found that pyrethroid insecticide-induced DNA damage may stem from enhanced lipid peroxidation and oxidative stress induced by excessive production of free radicals (particularly ROS), leading to redox imbalance. Zhou et al. [70] found that nanoplastics accumulated in earthworms and triggered ROS burst, inhibited antioxidant enzyme activity, and disrupted the lysosome-autophagy pathway, ultimately resulting in the death of coelomocyte immune cells; these toxic outcomes may stem from particle-derived physical effects as well as associated chemical contaminants.

### 6.2. Interference with DNA Damage Response Pathways

DDR is the core defense mechanism for maintaining genomic stability. Upon occurrence of different types of DNA damage, cells initiate highly specific repair processes. EEDs can act at multiple key nodes of DDR pathways, hindering proper damage recognition, signal transduction, and repair execution, thereby exacerbating genotoxicity and compromising genomic stability [104].

First, EEDs can interfere with early damage recognition and signal activation. They may inhibit the recruitment of the MRN complex, reduce the phosphorylation efficiency of ATM and ATR kinases, and decrease the formation of γH2AX foci, leading to ineffective sensing and transduction of DNA double-strand break signals [105,106]. Studies have shown that TDCPP and TCEP significantly upregulate ATM and γH2AX protein expression, which is both a marker of DNA double-strand breaks and a reflection of compensatory cellular responses to damage signals and interference with this signaling will directly affect the selection and initiation of downstream repair pathways [31,32].

Second, EEDs can disrupt cell cycle checkpoint regulation. They can downregulate core checkpoint proteins such as p53 and p21, leading to dysfunction of G1/S and G2/M checkpoints [107]. Notably, DNA repair mechanisms are closely related to the cell cycle. Homologous recombination (HR) mainly operates in the S/G2 phase, whereas non-homologous end joining (NHEJ) can function throughout the entire cell cycle [14]. Disruption of checkpoint function directly weakens the time-window effect of repair mechanisms.

Third, EEDs differentially inhibit repair pathways for different types of DNA damage. For DNA DSBs, DNA damage repair primarily relies on HR and NHEJ pathways [108]. Studies have found that many EEDs upregulate the mRNA expression of HR and NHEJ pathway genes at low concentrations, which appears to be a compensatory protective response [31,32]. However, at high concentrations or under prolonged exposure, some EEDs instead suppress key gene expression, shifting repair from high-fidelity HR to error-prone NHEJ, significantly increasing the risk of gene mutations and chromosomal translocations [107]. For single-strand breaks (SSBs) and base damage, cells activate three major pathways in coordination: base excision repair (BER), nucleotide excision repair (NER), and mismatch repair (MMR) [109]. EEDs with different chemical structures exhibit markedly different effects on these pathways. For example, the chlorinated organophosphate TDCPP tends to upregulate APEX1 and FEN1 genes (involved in BER for oxidative base modifications) but significantly downregulates MMR pathway genes (MSH2, MLH1) and NER pathway genes (DDB1, DDB2, ERCC1) [31]. The alkyl organophosphate TNBP activates most SSB repair pathways at lower concentrations but inhibits them at higher concentrations [32].

In addition, EEDs can also cross-regulate DDR through their endocrine-disrupting activities. Through hormone-mimicking, they can indirectly influence the transcriptional regulation of DDR-related genes, linking endocrine disruption with genotoxicity and forming a complex damage amplification loop [45]. For example, BPA exerts dual effects as both an estrogen agonist and an androgen antagonist [110]. By binding to estrogen receptors, it excessively promotes cell proliferation, inducing DNA replication stress and double-strand breaks. Simultaneously, it competitively inhibits the androgen receptor pathway, downregulating the transcription of core DDR genes such as ATM, BRCA1, and p53, thereby weakening DNA damage repair and cell cycle monitoring functions. TBT induced DNA strand breaks in fourth-instar Chironomus larvae at environmentally relevant concentrations and activated hormone nuclear receptor and early response gene expression [111]. In our previous study, we found that OPFRs can trigger GPER, activate PI3K/Akt and ERK signaling, change the expression of target proteins and genes associated with SSB and SDB damage-repair pathways, ultimately inducing DNA damage in GT1-7 cells [31,32]. Luo et al. [75] also revealed that three neonicotinoid insecticides induced DNA damage likely involving the PI3K-AKT signaling pathway and its crosstalk with other pathways. Hormonal disruption exacerbates DNA damage, while defects in repair pathways lead to the continued accumulation of genomic damage.

### 6.3. Disruption of Epigenetic Regulation

Unlike direct alterations of DNA sequence, epigenetic toxicity exerts indirect but long-lasting genetic hazards by modulating gene expression states. The relevant mechanisms mainly involve changes in DNA methylation, histone modifications, and non-coding RNA expression, which ultimately lead to aberrant silencing or activation of genes related to genomic stability [112].

EEDs can interfere with the activity and targeting of DNA methyltransferases, causing bidirectional disruption of global methylation levels. On one hand, they lead to hypermethylation of promoter regions of tumor suppressor genes and consequent transcriptional silencing, weakening the ability of cells to monitor proliferation [113,114]. On the other hand, they induce hypomethylation of promoters of proto-oncogenes or pro-proliferative genes (e.g., CCND1, BCL2) and aberrant activation, driving abnormal cell proliferation. Phthalates such as BBP, MBP, and MBzP significantly decreased global DNA methylation levels, induced hypermethylation of the promoter regions of tumor suppressor genes TP53 and P16 with consequent downregulation of their expression, and caused hypomethylation of the promoters of proto-oncogenes CCND1 and BCL2 with consequent upregulation [77]. This bidirectional dysregulation collectively creates a molecular environment conducive to uncontrolled proliferation and genomic instability, amplifying genotoxic insults and potentially increasing carcinogenic risks in human cells. In addition to DNA methylation, EEDs can also affect the activity of histone acetyltransferases, methyltransferases, and other modifying enzymes, changing the post-translational modification status of histones (acetylation, methylation, phosphorylation, etc.), thereby modulating chromatin accessibility in regions containing DNA damage repair genes [115]. This can indirectly reduce the transcription efficiency of repair proteins and weaken the capacity of cells for damage recognition and repair. Additionally, EEDs can also change the expression profiles of non-coding RNAs such as microRNAs (miRNAs), long non-coding RNAs (lncRNAs), and other non-coding RNAs [116,117]. These non-coding RNAs directly target and suppress the translation of mRNAs that encode DNA repair proteins or cell cycle checkpoint proteins. This post-transcriptional regulation further reduces the overall efficacy of the DDR network. Notably, the three epigenetic mechanisms do not act alone but work together. DNA methylation and histone modifications jointly regulate chromatin structure, while non-coding RNAs can target and modulate the expression of methyltransferases and histone-modifying enzymes [112]. Together, they form a multi-layered regulatory network that amplifies epigenetic toxicity, allowing EED-induced gene expression abnormalities to persist through cell divisions [113]. Such persistence is a key basis for long-term genotoxicity.

In addition, three core mechanisms can interact with each other and amplify progressively [118,119]. Specifically, excessive ROS accumulation overwhelms antioxidant defenses, leading to oxidative damage of lipids, proteins, and nucleic acids. This damage further impairs DDR efficiency, allowing mutations to accumulate unchecked. Concurrently, epigenetic alterations silence critical tumor suppressor genes while activating oncogenic pathways. Ultimately, the synergy among oxidative stress, defective DNA repair, and epigenetic shifts leads to accumulation of DNA damage, chromosomal aberrations, and genomic instability.

### 6.4. Synergistic Effects of Combined Exposure

It is noteworthy that in real environments, EEDs rarely exist as single compounds but rather as complex mixtures of multiple pollutants. Recent studies have demonstrated that combined exposure often produces more potent genotoxicity than single substances [120]; even these effects were transmitted to the F1 and F3 generations [121]. Co-exposure of polyamide microplastics and TCPP in thick-shelled mussels at environmentally relevant concentrations synergistically induced oxidative stress via adsorption interactions, suggesting that co-exposure of EEDs poses a potentially more severe genotoxic threat to aquatic ecosystems than exposure to a single compound [122]. The combined exposure groups of BPA, dibutyl phthalate, and cadmium exhibited significantly higher cytotoxicity, oxidative damage, and DNA damage in HepG2 cells than single exposure groups in a 3 × 3 full-factorial design [123]. Radiation alone did not induce obvious DNA damage in MEF cells, but combined exposure with Cr(VI) significantly increased comet tail moments and γH2AX foci numbers, accompanied by S-phase cell cycle arrest, indicating a synergistic induction of DNA strand breaks. However, research on combined genotoxicity remains limited.

Different types of EEDs can act on various genotoxicity endpoints, such as oxidative stress, DNA repair, cell cycle regulation, and chromosome segregation, and through cross-talk and mutual amplification among pathways [39,124]. The combined toxicity may far exceed the simple arithmetic sum of individual exposures and the synergistic effects of combined exposure are manifested in three main aspects. First, different EEDs simultaneously promote ROS production via multiple routes, pushing intracellular oxidative stress beyond the upper limit of single substances, resulting in more severe oxidative DNA damage [125]. Second, multiple EEDs jointly target different nodes of DDR pathways, producing multiple inhibitory effects on the repair system, leading to a comprehensive decline in the ability of cells to repair various types of damage [118]. Third, epigenetic regulation becomes more complex under combined exposure. Different EEDs can synergistically disrupt DNA methylation and histone modification homeostasis, accelerating chromatin structural disorganization and gene expression abnormalities, thereby accelerating the accumulation of DNA damage, chromosomal aberrations, and gene mutations [126].

## 7. Summary and Perspectives

This study comprehensively reviews the research advances of the genotoxic effects and underlying molecular mechanisms of both classical and emerging EEDs using in vivo animal and in vitro cell models over the past fifteen years. The integrated literature analysis indicates that bisphenols, phthalates, organophosphate flame retardants, pyrethroid insecticides, and emerging pollutants can induce genotoxic effects including DNA damage, chromosomal aberrations, and micronucleus formation. The core mechanisms primarily involve genotoxic insults driven by ROS-mediated oxidative stress, interference with DDR pathways, and epigenetic dysregulation. Under realistic multi-pollutant coexposure scenarios, combined exposure can significantly amplify genotoxicity through synergistic effects. Notably, some novel substitutes such as BPS and BPF exhibit even higher genotoxicity than the parent compound in some models, highlighting the urgent need for more rigorous safety evaluations of substitutes.

Based on qualitative synthesis of in vitro and in vivo studies compiled in this narrative review together with consensus viewpoints from published expert toxicological reviews, existing genotoxicity evidence varies considerably across different environmental endocrine-disrupting chemicals. Bisphenol analogues and phthalates exhibit comparatively consistent genotoxicity-related observations across diverse in vitro cellular models and several in vivo animal studies reported in the literature [11]. Negative genotoxic outcomes are occasionally reported in the literature, yet a substantial number of reported genotoxic endpoints are induced under experimental concentrations exceeding realistic environmentally relevant human exposure levels [127]. Notably, several key genotoxic phenotypes of classical EEDs have been replicated across independent experimental systems, genotoxic datasets for many emerging contaminants remain heterogeneous and lack cross-model validation [27,28].

Although numerous studies have confirmed the significant genotoxic potential of various EEDs, several research gaps and challenges remain that warrant future attention. First, the exposure scenarios in current studies deviate considerably from real environments. Most existing studies employ short-term, high-concentration exposure regimens, whereas actual environmental EED levels are typically low but persist over extended periods. Importantly, genotoxic effects observed at micromolar-range in vitro experimental concentrations should not be directly extrapolated to human health risks under real-world environmental exposure scenarios. Future efforts should prioritize the development of highly sensitive detection techniques and focus on the long-term health impacts of environmentally relevant low-dose exposures, so as to address the uncertainty associated with high-to-low dose extrapolation in current risk assessments.

Second, the research perspective needs to shift from single-compound to combined exposure. Most published work has centered on individual EEDs, while real environments typically harbor complex mixtures of multiple pollutants over prolonged timescales. Therefore, combined exposure more closely reflects real environmental exposure scenarios. Future studies should pay greater attention to the synergistic toxic effects of combined EED exposure under realistic coexposure scenarios to accurately assess genotoxicity risk. More importantly, combined exposure of multiple EEDs can break through the low-dose effect thresholds of single substances and induce more significant genetic damage even at extremely low environmental concentrations [39,128]. Therefore, the synergistic effects of combined exposure are a critical and indispensable aspect of EED genotoxicity.

Third, mechanistic investigations should be upgraded from fragmented to systematic integration. With the rapid advancement of computational toxicology, high-throughput screening technologies, multi-omics, and CRISPR gene editing, genotoxicity research is progressively moving from single-endpoint detection toward in-depth mechanistic dissection and multidimensional data integration. In recent years, artificial intelligence-based toxicity prediction models have begun to be applied to EED genotoxicity screening, opening new insights for efficient risk assessment of large chemical inventories. Future work should integrate these technological advantages to establish a tripartite research framework of “high-throughput in vitro screening, targeted in vivo validation, and computational modeling and prediction.”

In summary, the integration of high-throughput screening, multi-omics, and AI-based modeling should be further leveraged to focus on the genotoxic effects and transgenerational mechanisms under low-dose, long-term, and combined exposure scenarios. These efforts will help refine detection standards and risk assessment frameworks for EEDs, thereby providing a more robust scientific basis for environmental pollution control and the protection of human health.

## Figures and Tables

**Table 2:** MIE, KE and AO of genotoxicity triggered by typical EEDs across various test models.

Cell Lines/ Organisms	Type	Molecular Initiating Event (MIE)	Key Events (KE)	Adverse Outcome (AO)	Reference
Goodea atripinnis	BPA	BPA specifically binds to the FOXL2 transcription factor protein	Foxl2/CYP19A expression abnormalities; imbalance in estrogen andandrogen synthesis; DNA damage in the gonads	Impaired Reproductive Health	[8]
Cyprinus carpio	BPA	BPA accumulates in the tissues of multiple organs and disrupts the mitochondrial electron transport chain	Decreased antioxidant enzyme activity and increased accumulation Of 8-OHdG; double-strand breaks in DNA in brain, liver, gill, and kidney tissues; histopathological damage and abnormal blood biochemical markers	Multi-organ dysfunction	[92]
Daphnia magna	BPA	BPA penetrates the cell membranes of large copepod cells and damages the mitochondria within the cells.	Decreased antioxidant enzyme activity, increased 8-OHdG; widespread somatic DNA damage	Growth retardation, reduced reproductive capacity of the population	[93]
Mouse Immune Cells	BPA	BPA acts on macrophage membranes and mitochondria, inducing aburst of ROS and a cascade of oxidative stress reactions	Decreased antioxidant Enzyme activity and accumulation of 8-OHdG; phosphorylation of the p53 protein activates the p53 pathway; synergistic activation of the Ras/Raf/ERK pathway; activation of caspases	Macrophage death, liver tissue lesions	[94]
Zebrafish liver cells	BPs	Bisphenol analogs bind to live nuclear receptors and DNA repair-related proteins, damaging liver mitochondria and increasing ROS levels inhepatocytes.	Excessive ROS production inhepatocytes, decreased antioxidantenzyme activity, increased 8-OHdG; p53 phosphorylation, activation of thep53 pathway; activation of the as/Raf/ERK pathway, activation of caspases	Liver tissue lesions; somatic DNA damage leading to developmental/reproductive abnormalities	[95]
Gobiocypris rarus	CBZ	Carbamazepine Is metabolized by liver cells to form oxidative intermediates, Which damage mitochondria And trigger oxidative stress In liver cells.	Decreased antioxidant enzyme activity and accumulation of8-OHdG; oxidative damage and DNA breaks in hepatocytes; activation of the p53 and Ras/Raf/ERK apoptotic pathways; and activation of caspases	Liver tissue lesions, multi-organdysfunction, andreduced reproductive capacity	[96]
Cyprinuscarpio	BPA	BPA crosses the blood-brain barrier and damages neuronal mitochondria, leading to the Massive release of ROS in brain tissue	Decreased antioxidant enzyme activity and elevated 8-OHdG levels in brain tissue; structural damage to the blood–brain barrier; downregulation of the brain immune factors IgM and NO	Neurologicaldysfunction	[97]

## Data Availability

No new data were created or analyzed in this study. Data sharing is not applicable to this article.

## Supplementary Materials

The following supporting information can be downloaded at: https://www.mdpi.com/article/10.3390/toxics14100882/s1, Table S1: Summary of in vivo genotoxicity data of typical environmental endocrine disruptors (animal models); Table S2: Summary of in vitro genotoxicity data of typical environmental endocrine disruptors (human cell lines).

## Appendix A

**Table A1:** Environmental exposure levels of typical environmental endocrine disruptors in surface-water, wastewater and sediment.

Source Category	Compound	Surface Water	Wastewater	Sediment	Reference
Industrial sources	Bisphenol analogues	ND(no detection)-hundreds ng/L	ng/L-low μg/L	nd-hundreds ng/g dw	[129,130,131]
	Phthalate esters	tens ng/L-several μg/L	hundreds ng/L-several μg/L	tens-hundreds ng/g dw	[132,133]
	PBDEs	pg-low ng/L in dissolved phase	ng/L-low hundreds ng/L	ng/g-several μg/g dw	[134,135]
Consumer-product-derived	Nonylphenols (NPs)	ND-several μg/L	Influent: ND-tens μg/L	ND-low hundreds μg/g dw	[136]
	Triclosan (TCS)	ND-hundreds ng/L	ND-several μg/L in influent	ND-several μg/g dw	[137]
	Parabens (MeP, EtP, PrP, BuP)	ND-several hundred ng/L	ND-several thousand ng/L (influent)	ND-low hundreds ng/g dw	[138]
Combustion-/transformation-derived products	6PPD-quinone	ND-hundreds ng/L; sharp concentration spikes in road-impacted stormwater runoff	ND-hundreds ng/L in urban road runoff; limited municipal wastewater monitoring data	-	[26]
Emerging contaminants	PFAS (PFOA, PFOS)	ND-several hundred ng/L	ND-low μg/L	(Variable; long-chain homologues preferentially associate with solid-phase matrices)	[139]
	Micro-/nanoplastics	Variable abundance; elevated in urban-impacted catchments; dominated by fibers and fragments	Influent: high particle abundance; partial removal during wastewater treatment; residual particles persist in effluents	-	[140]

## References

[1] Robles-Matos N., Artis T., Simmons R.A., Bartolomei M.S. (2021). Environmental exposure to endocrine disrupting chemicals influences genomic imprinting, growth, and metabolism. Genes.

[2] Bagade S., Verma K., Choudhary K., Khan D. (2026). Persistent organic pollutants in plastics: A historical and sector specific review from an Indian environmental health perspective. Water Air Soil Pollut..

[3] Onyime S.C., Nworie F.S. (2026). Endocrine-disrupting chemicals: Environmental impact, exposure pathways, and human health risks—A review. Chem. Afr..

[4] Sánchez-Ocaña M., Ruiz de Porras V. (2025). Environmental endocrine disruptors and breast cancer: The role of bisphenols, polychlorinated biphenyls, parabens, and dioxins. Environ. Toxicol. Pharmacol..

[5] Aquilino M., Sánchez-Argüello P., Martínez-Guitarte J.-L. (2018). Genotoxic effects of vinclozolin on the aquatic insect *Chironomus riparius* (*Diptera, Chironomidae*). Environ. Pollut..

[6] Bridgeman L., Juan C., Berrada H., Severin I., Juan-García A. (2025). Evaluating the genotoxicity and mutagenicity of good contaminants: Acrylamide, penitrem A, and 3-acetyldeoxynivalenol in individual and combined exposure in vitro. J. Appl. Toxicol..

[7] Bergman Å., Heindel J., Jobling S., Kidd K., Zoeller R.T. (2012). State-of-the-science of endocrine disrupting chemicals, 2012. Toxicol. Lett..

[8] Cervantes-Camacho I., Guerrero-Estévez S.M., López M.F., Alarcón-Hernández E., López-López E. (2020). Effects of bisphenol A on Foxl2 gene expression and DNA damage in adult viviparous fish *Goodea atripinnis*. J. Toxicol. Environ. Health Part A.

[9] Frenzilli G., Martorell-Ribera J., Bernardeschi M., Scarcelli V., Jönsson E., Diano N., Moggio M., Guidi P., Sturve J., Asker N. (2021). Bisphenol A and bisphenol S induce endocrine and chromosomal alterations in brown trout. Front. Endocrinol..

[10] Kose O., Rachidi W., Beal D., Erkekoglu P., Fayyad-Kazan H., Gumusel B.K. (2020). The effects of different bisphenol derivatives on oxidative stress, DNA damage and DNA repair in RWPE-1 cells: A comparative study. J. Appl.Toxicol..

[11] Dhar S., Tomar A., Anupama N., Chatterjee P., Chatterjee P.K. (2026). Phthalates as the silent saboteurs of male fertility via changes in semen quality: A systematic review. Reprod. Biol. Endocrinol..

[12] Vasconcelos A.L., Silva M.J., Louro H. (2019). In vitro exposure to the next-generation plasticizer diisononyl cyclohexane-1,2-dicarboxylate (DINCH): Cytotoxicity and genotoxicity assessment in human cells. J. Toxicol. Environ. Health A.

[13] Wang L., Murtala N.M., Hu K.Q., Chen Y.J., Chen M.X., Sun H.T., Liu Y.G. (2024). Prediction of polybrominated diphenyl ethers (PBDEs) as potential substrates of various human CYP enzymes and laboratory test of BDE-99 for its metabolism-activated mutagenicity. Toxicology.

[14] Huang P.X., Yang J., Ning J., Wang M., Song Q.S. (2015). Atrazine triggers DNA damage response and induces DNA double-strand breaks in MCF-10A cells. Int. J. Mol. Sci..

[15] Lopes A.T.d.C., Benvindo-Souza M., Sotero D.F., Pedroso T.M.A., Tomaz A.A., Costa A.G., Gois A.R.d.S., Vieira T.B., Bastos R.P., Silva D.d.M.e. (2025). Sublethal effects of atrazine concentrations exposure on tadpoles of *Dendropsophus minutus*: Evaluation of redox status, micronuclei frequencies and comet assay as biomarkers. Aquat. Toxicol..

[16] Yu R.Y., Zhou Y., Xu S.X., Jing J., Zhang H.Y., Huang Y.F. (2023). Distribution, transfer, and health risk of organochlorine pesticides in soil and water of the Huangshui River basin. Toxics.

[17] Mohafrash S.M.M., Hassan E.E., El-shaer N.H., Mossa A.T.H. (2021). Detoxification gene expression, genotoxicity, and hepatorenal damage induced by subacute exposure to the new pyrethroid, imiprothrin, in rats. Environ. Sci. Pollut. Res..

[18] Bernardi F., Lirola J.R., Cestari M.M., Bombardelli R.A. (2022). Effects on reproductive, biochemical and genotoxic parameters of herbicides 2,4-D and glyphosate in silver catfish (*Rhamdia quelen*). Environ. Toxicol. Pharmacol..

[19] Rosculet C.A., Bonciu E., Rosculet E., Olaru L.A. (2019). Determination of the environmental pollution potential of some herbicides by the assessment of cytotoxic and genotoxic effects on *Allium cepa*. Int. J. Environ. Res. Public Health.

[20] Noorimotlagh Z., Mirzaee S.A., Ahmadi M., Jaafarzadeh N., Rahim F. (2018). The possible DNA damage induced by environmental organic compounds: The case of nonylphenol. Ecotoxicol. Environ. Saf..

[21] Song C.F., Lin J., Huang X.L., Wu Y.H., Liu J.T., Wu C.X. (2016). Effect of butyl paraben on the development and microbial composition of periphyton. Ecotoxicology.

[22] Hawash H.B., Moneer A.A., Galhoum A.A., Elgarahy A.M., Mohamed W.A.A., Samy M., El-Seedi H.R., Gaballah M.S., Mubarak M.F., Attia N.F. (2023). Occurrence and spatial distribution of pharmaceuticals and personal care products (PPCPs) in the aquatic environment, their characteristics, and adopted legislations. J. Water Process Eng..

[23] Oropesa A.L., Floro A.M., Palma P. (2016). Assessment of the effects of the carbamazepine on the endogenous endocrine system of Daphnia magna. Environ. Sci. Pollut. Res..

[24] Li J.B., Zhang H.F., Han Y.N., Huang C., Ma M., Yang M. (2020). Cytotoxicity and genotoxicity assays of halobenzoquinones disinfection byproducts using different human cell lines. Environ. Mol. Mutagen..

[25] Pidoux G., Gerbaud P., Guibourdenche J., Thérond P., Ferreira F., Simasotchi C., Evain-Brion D., Gil S. (2015). Formaldehyde Crosses the Human Placenta and Affects Human Trophoblast Differentiation and Hormonal Functions. PLoS ONE.

[26] Wang S., Xu S.H., Lu J.H., Wan Y.S., Kang Z.Y., Chen H., Islam S., Gao B. (2026). Occurrence, transformation, and toxicity of tire-derived chemicals 6PPD and 6PPD-q in the Environment. Environ. Sci. Technol..

[27] Ullah S., Ahmad S., Guo X., Ullah S., Ullah S., Nabi G., Wanghe K. (2023). A review of the endocrine disrupting effects of micro and nano plastic and their associated chemicals in mammals. Front. Endocrinol..

[28] Ojo A.F., Peng C., Ng J.C. (2022). Genotoxicity assessment of per- and polyfluoroalkyl substances mixtures in human liver cells (HepG2). Toxicology.

[29] An J., Hu J.W., Shang Y., Zhong Y.F., Zhang X.Y., Yu Z.Q. (2016). The cytotoxicity of organophosphate flame retardants on HepG2, A549 and Caco-2 cells. J. Environ. Sci. Health Part A.

[30] Pyambri M., Jaumot J. (2025). Toxicity Assessment of Organophosphate Flame Retardants Using New Approach Methodologies. Toxics.

[31] Lei B.L., Li J.Y., Yang Y.X., Lu X.Y., Liu T.Y., Li N., Li Y., Li X.Y., Feng C.L. (2026). Mechanistic insights into TDCPP-induced DNA damage in GT1-7 cells. Toxicology.

[32] Lei B.L., Lu X.Y., Yang Y.X., Li J.Y., Zhong Y.F., Zhang X.L., Li J. (2025). Effects of two typical organophosphate flame retardants on DNA damage of GT1-7 cells and relevant molecular mechanism. Toxicology.

[33] Hayashi M. (2022). Opinion: Regulatory genotoxicity: Past, present and future. Genes Environ..

[34] Zhang R.Y., Yang J., Liu X. (2011). Ecotoxic effects and genotoxicity of pentachlorophenol. Chin. Environ. Chem..

[35] Collins A.R. (2004). The Comet Assay for DNA damage and repair: Principles, applications, and limitations. Mol. Biotechnol..

[36] Pribakovic R., Bornhorst J., Stopper H., Bankoglu E.E. (2025). Influence of pH and lysis duration on DNA damage detection: A comparison of neutral and alkaline comet assays. Mutagenesis.

[37] Møller P., Azqueta A., Boutet-Robinet E., Koppen G., Bonassi S., Milic M., Gajski G., Costa S., Teixeira J.P., Pereira C.C. (2020). Minimum information for reporting on the comet assay (MIRCA): Recommendations for describing comet assay procedures and results. Nat. Protoc..

[38] Lundqvist J., Hellman B., Oskarsson A. (2016). Fungicide prochloraz induces oxidative stress and DNA damage in vitro. Food Chem. Toxicol..

[39] Karzi V., Ozcagli E., Tzatzarakis M.N., Vakonaki E., Fragkiadoulaki I., Kalliantasi I., Chalkiadaki C., Alegakis A., Stivaktakis P., Karzi A. (2023). DNA Damage estimation after chronic and combined exposure to endocrine disruptors: An in vivo real-life risk simulation approach. Int. J. Mol. Sci..

[40] Dantas F.G.d.S., de Castilho P.F., de Almeida-Apolonio A.A., de Araújo R.P., de Oliveira K.M.P. (2020). Mutagenic potential of medicinal plants evaluated by the *Ames Salmonella*/microsome assay: A systematic review. Mutat. Res. Rev. Mutat. Res..

[41] Bhawsinghka N., Schaaper R.M. (2026). Detection of mutations: From *Ames* test to duplex sequencing. Front. Mol. Biosci..

[42] Cheng C., Fei Z., Xiao P.F. (2023). Methods to improve the accuracy of next-generation sequencing. Front. Bioeng. Biotechnol..

[43] Fenech M. (2000). The in vitro micronucleus technique. Mutat. Res..

[44] Heylmann D., Kaina B. (2016). The γH2AX DNA damage assay from a drop of blood. Sci. Rep..

[45] Mahemuti L., Chen Q., Coughlan M.C., Qiao C., Chepelev N.L., Florian M., Dong D., Woodworth R.G., Yan J., Cao X. (2018). Bisphenol A induces DSB-ATM-p53 signaling leading to cell cycle arrest, senescence, autophagy, stress response, and estrogen release in human fetal lung fibroblasts. Arch. Toxicol..

[46] Sibiya A., Al-Ghanim K.A., Govindarajan M., Nicoletti M., Sachivkina N., Vaseeharan B. (2023). Biochemical patterns and genotoxicity of the endocrine disruptor metformin in the freshwater fish *Labeo rohita*. Fishes.

[47] Xu X.L., He Y.L., Cheng Z.F., Zhang H.Y., Chu Y.J., Wang Z.W., An X.P. (2024). Environmental endocrine disrupting chemical-DEHP exposure-provoked biotoxicity about microbiota-gut-mammary axis in lactating mice *via* multi-omics technologies. Environ. Int..

[48] Shi C.C., Cheng L., Yu Y., Chen S.S., Dai Y.B., Yang J.J., Zhang H.J., Chen J.P., Geng N.B. (2024). Multi-omics integration analysis: Tools and applications in environmental toxicology. Environ. Pollut..

[49] Wang J., Hong X.S., Liu W., Zhang L., Yan S.H., Li Z.T., Zha J.M. (2025). Comprehensive assessment of the safety of bisphenol A and its analogs based on multi-toxicity tests in vitro. J. Hazard. Mater..

[50] Lynch C., Sakamuru S., Ooka M., Huang R., Klumpp-Thomas C., Shinn P., Gerhold D., Rossoshek A., Michael S., Casey W. (2024). High-throughput screening to advance in vitro toxicology: Accomplishments, challenges, and future directions. Annu. Rev. Pharmacol. Toxicol..

[51] Mortelmans K., Zeiger E. (2000). The Ames Salmonella/microsome mutagenicity assay. Mutat. Res..

[52] Organisation for Economic Co-Operation and Development (2016). Test No. 490: In Vitro Mammalian Cell Gene Mutation Tests Using the Thymidine Kinase Gene.

[53] Thybaud V., Dean S., Boerrigter M., Casciano D., Douglas G.R., Glickman B.W., Gorelick N.J., Mirsalis J.C., Martus H.J., Skopek T.R. (2003). In vivo transgenic mutation assays. Environ. Mol. Mutagen..

[54] Schmitt M.W., Kennedy S.R., Salk J.J., Fox E.J., Hiatt J.B., Reid-Bayliss K.S., True L.D., Loeb L.A. (2012). Detection of ultra-rare mutations by next-generation sequencing. Proc. Natl. Acad. Sci. USA.

[55] Kopp B., Khoury L., Audebert M., Zalko D. (2019). Validation of the γH2AX biomarker for genotoxicity assessment: Strengths, pitfalls and perspectives. Arch. Toxicol..

[56] Bryce S.M., Bernacki D.T., Bemis J.C., Avlasevich S.L., Dertinger S.D. (2016). Genotoxic mode of action predictions from a multiplexed flow cytometric assay and a machine-learning approach. Environ. Mol. Mutagen..

[57] Cho E., Allemang A., Audebert M., Chauhan V., Dertinger S., Hendriks G., Luijten M., Marchetti F., Minocherhomji S., Pfuhler S. (2022). AOP report: Development of an adverse outcome pathway for oxidative DNA damage leading to mutations and chromosomal aberrations. Environ. Mol. Mutagen..

[58] Allemang A., De Abrew K.N., Shan Y.K., Krailler J.M., Pfuhler S. (2021). A comparison of classical and 21st century genotoxicity tools: A proof of concept study of 18 chemicals comparing in vitro micronucleus, ToxTracker and genomics-based methods (TGx-DDI, whole genome clustering and connectivity mapping). Environ. Mol. Mutagen..

[59] Godschalk R., Faulk C., LaRocca J., van Benthem J., Marchetti F. (2025). Epigenotoxicity: Decoding the epigenetic imprints of genotoxic agents and their implications for regulatory genetic toxicology. Environ. Mol. Mutagen..

[60] Wu H.J., Liu C., Duan W.X., Xu S.C., He M.D., Chen C.H., Wang Y., Zhou Z., Yu Z.P., Zhang L. (2013). Melatonin ameliorates bisphenol A-induced DNA damage in the germ cells of adult male rats. Mutat. Res.-Genet. Toxicol. Environ. Mutagen..

[61] Laio T.Y., Chen C.C., Tsou H.H., Liu T.Y., Wang H.T. (2019). Acute and chronic exposure of toluene induces genotoxicity in different regions of the brain in normal and allergic mouse models. Neurotox. Res..

[62] Martínez-Paz P., Morales M., Martínez-Guitarte J.L., Morcillo G. (2013). Genotoxic effects of environmental endocrine disruptors on the aquatic insect *Chironomus riparius* evaluated using the comet assay. Mutat. Res..

[63] Vincze K., Gehring M., Braunbeck T. (2014). (Eco)toxicological effects of 2,4,7,9-tetramethyl-5-decyne-4,7-diol (TMDD) in zebrafish (*Danio rerio*) and permanent fish cell cultures. Environ. Sci. Pollut. Res..

[64] Du Y.T., Yu J.J., Huang G., Zhang K., Abdelhafez H.E.D.H., Yin X.H., Qiao J.K., Guo J.F. (2021). Regulation of TRAF6 by microRNA-146a in zebrafish embryos after exposure to di(2-ethylhexyl) phthalate at different concentrations. Chem. Res. Toxicol..

[65] Rivero-Wendt C.L.G., Miranda-Vilela A.L., Ferreira M.F.N., Amorim F.S., da Silva V.A.G., Louvandini H., Grisolia C.K. (2013). Lack of genotoxicity in *Astyanax bimaculatus* and *Oreochromis niloticus* of 17α-methyltestosterone used in fish hatcheries to produce male monosex populations. Genet. Mol. Res..

[66] Hussain R., Mahmood F., Khan M.Z., Khan A., Muhammad F. (2011). Pathological and genotoxic effects of atrazine in male Japanese quail (*Coturnix japonica*). Ecotoxicology.

[67] Zakariah M., Molele R.A., Mahdy M.A.A., Ibrahim M.I.A., McGaw L.J. (2022). Structural observations on spermatogenic cells of Japanese quail (*Coturnix coturnix japonica*) pre-pubertally exposed to dibutyl phthalate: A light and transmission electron microscopy study. Micron.

[68] Scaia M.F., de Gregorio L.S., Franco-Belussi L., Succi-Domingues M., de Oliveira C. (2019). Gonadal, body color, and genotoxic alterations in *Lithobates catesbeianus* tadpoles exposed to nonylphenol. Environ. Sci. Pollut. Res..

[69] Chen L.Y., Bai J., Wan J., Song Y., Xiang G.H., Duan R.Y., Zheng Y. (2024). Endocrine system, cell growth and death, and energy metabolism induced by Sb(III) exposure in earthworm (*Pheretima guillemi*) revealed by transcriptome and metabolome analysis. Environ. Pollut..

[70] Zhou Y.F., He G., Jiang H., Pan K., Liu W.Z. (2023). Nanoplastics induces oxidative stress and triggers lysosome-associated immune-defensive cell death in the earthworm *Eisenia fetida*. Environ. Int..

[71] Weissman I.L. (2000). Translating stem and progenitor cell biology to the clinic: Barriers and opportunities. Science.

[72] Schmidt J., Kotnik P., Trontelj J., Knez Ž., Mašič L.P. (2013). Bioactivation of bisphenol A and its analogs (BPF, BPAF, BPZ and DMBPA) in human liver microsomes. Toxicol. Vitr..

[73] Kara-Ertekin S., Yazar S., Erkan M. (2021). In vitro toxicological assessment of flumethrin’s effects on MCF-7 breast cancer cells. Hum. Exp. Toxicol..

[74] Al-Saleh I., Al-Rajudi T., Al-Qudaihi G., Manogaran P. (2017). Evaluating the potential genotoxicity of phthalates esters (PAEs) in perfumes using in vitro assays. Environ. Sci. Pollut. Res..

[75] Luo F. (2018). Study on Genotoxicity and Mechanism of Typical Neonicotinoid Insecticides in Human Bone Marrow Mesenchymal Stem Cells. Master’s Dissertation.

[76] Duan C.E., Gong B., Yang X.B. (2014). In vitro genotoxicity of poly(butylene succinate) extract toward human peripheral blood mononuclear cells. Chin. J. Tissue Eng. Res..

[77] Mokra K., Kuźmińska-Surowaniec A., Woźniak K., Michałowicz J. (2017). Evaluation of DNA-damaging potential of bisphenol A and its selected analogs in human peripheral blood mononuclear cells (in vitro study). Food Chem. Toxicol..

[78] Sicińska P., Jabłońska E., Bukowska B., Balcerczyk A., Reszka E. (2022). The selected epigenetic effects of phthalates: DBP, BBP and their metabolites: MBP, MBzP on human peripheral blood mononuclear cells (in vitro). Toxicol. Vitr..

[79] Kang Y.H., Jeong H.J., Park Y.J. (2024). Hydramethylnon induces mitochondria-mediated apoptosis in BEAS-2B human bronchial epithelial cells. Toxicol. Appl. Pharmacol..

[80] Ichikawa M., Shimokawa N., Makuta M., Song Y., Yao J., Dolenc D., Kononenko V., Drobne D., Bohinc K., Malev O. (2025). Cytotoxicity, genotoxicity, and enzyme activity effects of UV filters and their chlorinated by-products in human lung epithelial cells (A549). Sci. Total Environ..

[81] Damdam S., Hunnie B., Jones P., Hecker M., Brinkmann M., da Silva Junior F.C. (2026). Toxicity assessment of 6PPD-quinone in human lung cells: Insights from BEAS-2B and A549 cell lines. Toxicol. Vitr..

[82] Pérez-Albaladejo E., Pinteño R., Aznar-Loque M.C., Casado M., Postigo C., Porte C. (2023). Genotoxicity and endocrine disruption potential of haloacetic acids in human placental and lung cells. Sci. Total Environ..

[83] Blanc M., Rüegg J., Scherbak N., Keiter S.H. (2019). Environmental chemicals differentially affect epigenetic-related mechanisms in the zebrafish liver (ZF-L) cell line and in zebrafish embryos. Aquat. Toxicol..

[84] Siqueira P.R., do Carmo T.L.L., Bonomo M.M., dos Santos F.A., Fernandes M.N. (2021). Proliferative response avoids mutagenic effects of titanium dioxide (TiO_2_) nanoparticles in a zebrafish hepatocyte cell line. J. Hazard. Mater. Adv..

[85] Monsinjon T., Knigge T. (2025). Endocrine disrupters affect the immune system of fish: The example of the European seabass. Fish Shellfish Immunol..

[86] Sponchiado G., Reynaldo E.M.F.L., Andrade A.C.B., Vasconcelos E.C., Adam M.L., Oliveira C.M.R. (2011). Genotoxic effects in erythrocytes of *Oreochromis niloticus* exposed to nanograms-per-liter concentration of 17β-estradiol (E_2_): An assessment using micronucleus test and comet assay. Water Air Soil Pollut..

[87] Xin L.L., Lin Y., Wang A.Q., Zhu W., Liang Y., Su X.J., Hong C.J., Wan J.M., Wang Y.R., Tian H.L. (2015). Cytogenetic evaluation for the genotoxicity of bisphenol-A in Chinese hamster ovary cells. Environ. Toxicol. Pharmacol..

[88] Zhu Y., Zhu L.T., Lan Y., Sun C., Chen G.D. (2026). Exposure to hexavalent chromium and 1800 MHz electromagnetic radiation can synergistically induce intracellular DNA damage in mouse embryonic fibroblasts. Biochem. Biophys. Res. Commun..

[89] Fujitani K., Otomo A., Wada M., Takamatsu N., Ito M. (2016). Sexually dimorphic expression of Dmrt1 and γH2AX in germ stem cells during gonadal development in *Xenopus laevis*. FEBS Open Bio..

[90] Hyakutake K., Kawasaki T., Zhang J.D., Kubota H., Abe S.-I., Takamune K. (2015). A symmetrical allocation of JAK1 mRNA during spermatogonial stem cell division in *Xenopus laevis*. Dev. Growth Differ..

[91] Ma T.T., Chen L.K., Wu L.H., Zhang H.B., Luo Y.M. (2016). Oxidative stress, cytotoxicity and genotoxicity in earthworm *Eisenia fetida* at different di-n-butyl phthalate exposure levels. PLoS ONE.

[92] Afzal G., Ahmad H.I., Hussain R., Jamal A., Kiran S., Hussain T., Saeed S., Nisa M.U. (2022). Bisphenol A induces histopathological, hematobiochemical alterations, oxidative stress, and genotoxicity in common carp (*Cyprinus carpio* L.). Oxid. Med. Cell. Longev..

[93] Sreevidya C.P., Manoj Kumar T.M., Soumya B., Suresh K., Manomi S., Magnuson J.T., Puthumana J. (2025). Establishment of a cell culture from *Daphnia magna* as an in vitro model for (eco)toxicology assays: Case study using bisphenol A as a representative cytotoxic and endocrine disrupting chemical. Aquat. Toxicol..

[94] Huang F.M., Chang Y.C., Lee S.S., Ho Y.C., Yang M.L., Lin H.W., Kuan Y.H. (2018). Bisphenol A exhibits cytotoxic or genotoxic potential via oxidative stress-associated mitochondrial apoptotic pathway in murine macrophages. Food Chem. Toxicol..

[95] Štern A., Kološa K., Rozman Š., Žegura B. (2025). Exploring the genotoxic potential of bisphenol A and its emerging alternatives in an advanced in vitro 3D zebrafish hepatic cell model. Sci. Total Environ..

[96] Yan S.H., Chen R., Wang M., Zha J.M. (2021). Carbamazepine at environmentally relevant concentrations caused DNA damage and apoptosis in the liver of Chinese rare minnows (*Gobiocypris rarus*) by the Ras/Raf/ERK/p53 signaling pathway. Environ. Pollut..

[97] Tahir R., Samra, Afzal F., Khan H., Ghaffar A., Qazi I.H., Al-Khattaf F.S., Zhao L., Yan H., Kuo H. (2024). Chronic bisphenol A induced neurotoxicity: Exposure risk, molecular fate within carp and its potential phytoremediation. Sci. Total Environ..

[98] Şahiner H.S., Ak M.O., Bardakçı Yilmaz Ö., Avcıoğlu M., Boyacioğlu M., Kum C., Sekkin S. (2026). Comparative dose-dependent effects of bisphenol A, F, and S on testicular oxidative stress, DNA damage, and reproductive hormones in rats. J. Appl. Toxicol..

[99] Oya-Silva L.F., Guiloski I.C., Vicari T., Deda B., Marcondes F.R., Simeoni R.D., Perussolo M.C., Martino-Andrade A.J., Leme D.M., de Assis H.C.S. (2023). Evidence of genotoxicity, neurotoxicity, and antioxidant imbalance in silver catfish *Rhamdia quelen* after subchronic exposure to diisopentyl phthalate. Mutat. Res.-Genet. Toxicol. Environ. Mutagen..

[100] Mukherjee U., Ghosh S., Maitra S. (2025). Multi-mechanistic effects of bisphenol A on testicular dysfunction and endocrine disruption in adult male *Labeo bata*: Oxidative stress, inflammation, and dysregulated energy sensors. Environ. Pollut..

[101] Costa-Silva D.G., Leandro L.P., de Brum Vieira P., de Carvalho N.R., Lopes A.R., Schimith L.E., Nunes M.E.M., de Mello R.S., Martins I.K., de Paula A.A. (2018). N-acetylcysteine inhibits Mancozeb-induced impairments to the normal development of zebrafish embryos. Neurotoxicol. Teratol..

[102] Gu Q., Silva C.S., Twaddle N.C., Beland F.A., Kanungo J. (2026). N-acetylcysteine prevents arsenic-induced apoptosis but not supernumerary motor neuron development in zebrafish embryos: Assessment of protein carbonylation and the p53 pathway. Int. J. Mol. Sci..

[103] Banik A., Eum J., Hwang B.J., Kee Y. (2023). Differential neuroprotective effects of N-acetylcysteine against dithianon toxicity in glutamatergic, dopaminergic, and GABAergic neurons: Assessment using zebrafish. Antioxidants.

[104] Ward A., Khanna K.K., Wiegmans A.P. (2015). Targeting homologous recombination, new pre-clinical and clinical therapeutic combinations inhibiting RAD51. Cancer Treat. Rev..

[105] Çavaş T., Çinkılıç N., Vatan Ö., Yılmaz D., Coşkun M. (2012). In vitro genotoxicity evaluation of acetamiprid in CaCo-2 cells using the micronucleus, comet and γH2AX foci assays. Pestic. Biochem. Physiol..

[106] Maréchal A., Zou L. (2013). DNA damage sensing by the ATM and ATR kinases. CSH Perspect. Biol..

[107] Elkafas H., Ali M., Elmorsy E., Kamel R., Thompson W.E., Badary O., Al-Hendy A., Yang Q. (2020). Vitamin D3 ameliorates DNA damage caused by developmental exposure to endocrine disruptors in the uterine myometrial stem cells of Eker Rats. Cells.

[108] Yedidia-Aryeh L., Goldberg M. (2022). The interplay between the cellular response to DNA double-strand breaks and estrogen. Cells.

[109] Tong J., Song J.W., Zhang W.C., Zhai J.B., Guan Q.L., Wang H.Q., Liu G.T., Zheng C.F. (2024). When DNA-damage responses meet innate and adaptive immunity. Cell. Mol. Life Sci..

[110] Lombó M., Herráez P. (2021). The effects of endocrine disruptors on the male germline: An intergenerational health risk. Biol. Rev..

[111] Morales M., Martínez-Paz P., Ozáez I., Martínez-Guitarte J.L., Morcillo G. (2013). DNA damage and transcriptional changes induced by tributyltin (TBT) after short in vivo exposures of *Chironomus riparius* (Diptera) larvae. Comp. Biochem. Physiol. C.

[112] Liang Y.J., Lu Q.S., Chen M.J., Zhao X.M., Chu C., Zhang C.F., Yuan J.H., Liu H.M., Lash G.E. (2025). Impact of endocrine disrupting chemicals (EDCs) on epigenetic regulation in the uterus: A narrative review. Reprod. Biol. Endocrin..

[113] Shi Y., Qi W., Xu Q., Wang Z., Cao X., Zhou L., Ye L. (2021). The role of epigenetics in the reproductive toxicity of environmental endocrine disruptors. Environ. Mol. Mutagen..

[114] Singh D.D. (2025). Epigenetic mechanisms of endocrine-disrupting chemicals in breast cancer and their impact on dietary intake. J. Xenobiot..

[115] Casati L., Sendra R., Sibilia V., Celotti F. (2015). Endocrine disruptors: The new players able to affect the epigenome. Front. Cell Dev. Biol..

[116] Mohammed A., Atkin S.L., Brennan E. (2025). Dysregulation of microRNA (miRNA) due to phthalate/phthalate metabolite exposure and associated health effects: A narrative review. J. Xenobiot..

[117] Yang Z., Xu F., Teschendorff A.E., Zhao Y., Yao L., Li J., He Y.A. (2022). Insights into the role of long non-coding RNAs in DNA methylation mediated transcriptional regulation. Front. Mol. Biosci..

[118] Zhang S., Chen H., Zhang J.N., Li J., Hou H.W., Hu Q.Y. (2020). The multiplex interactions and molecular mechanism on genotoxicity induced by formaldehyde and acrolein mixtures on human bronchial epithelial BEAS-2B cells. Environ. Int..

[119] Balabanič D., Filipič M., Krivograd Klemenčič A., Žegura B. (2021). Genotoxic activity of endocrine disrupting compounds commonly present in paper mill effluents. Sci. Total Environ..

[120] Zhang S., Chen H., Wang A., Liu Y., Hou H.W., Hu Q.Y. (2018). Combined effects of co-exposure to formaldehyde and acrolein mixtures on cytotoxicity and genotoxicity in vitro. Environ. Sci. Pollut. Res..

[121] Darracq-Ghitalla-Ciock M., Rajkumari N., Veyrenc S., Chuffart F., Attia S., Hiriart-Bryant E., Vial G., Rangarajan S., Tubbs E., Raveton M. (2025). Environmental exposure to endocrine disruptors induces insulin resistance and hepatic inflammation in adult male rats, with partial transmission across subsequent generations. Environ. Pollut..

[122] Chen Z.W., Huang W., Zhong Z., Khor W., Hu M.G., Wang Y.J. (2025). Beyond carrier effects: Polyamide microplastics and TCPP jointly drive physiological toxicity in mussels at environmental concentrations. Environ. Pollut..

[123] Li X.H., Yin P.H., Zhao L. (2017). Effects of individual and combined toxicity of bisphenol A, dibutyl phthalate and cadmium on oxidative stress and genotoxicity in HepG2 cells. Food Chem. Toxicol..

[124] Bachiri K., Kantar D., Laurent E.M.N., Gaboriaud P., Durand L., Drouin A., Chollot M., Schrama D., Houben R., Kervarrec T. (2025). DNA damage stress control is a truncated large T antigen and euchromatic histone lysine methyltransferase 2–dependent central feature of merkel cell carcinoma. J. Investig. Dermatol..

[125] Liu X., Han Z.Y., Han K.B., Pang Y.H., Zhao X.Y., Wang Y.T., Wu X.Y., Wang T.W. (2026). Oxidative stress, DNA damage, DNA repair inhibition, and apoptosis induced by lead and cadmium combined exposure in TK6 cells. Toxics.

[126] Lu X., Fraszczyk E., van der Meer T.P., van Faassen M., Bloks V.W., Kema I.P., van Beek A.P., Li S., Franke L., Westra H.-J. (2020). An epigenome-wide association study identifies multiple DNA methylation markers of exposure to endocrine disruptors. Environ. Int..

[127] Tsatsakis A.M., Docea A.O., Tsitsimpikou C. (2016). New challenges in risk assessment of chemicals when simulating real exposure scenarios; simultaneous multi-chemicals’ low-dose exposure. Food Chem. Toxicol..

[128] Rattan S., Flaws J.A. (2019). The epigenetic impacts of endocrine disruptors on female reproduction across generations. Biol. Reprod..

[129] Wan Y.J., Xia W., Yang S.Y., Pan X.Y., He Z.Y., Kannan K. (2018). Spatial distribution of bisphenol S in surface water and human serum from Yangtze River watershed, China: Implications for exposure through drinking water. Chemosphere.

[130] Lalwani D., Ruan Y.F., Taniyasu S., Yamazaki E., Kumar N.J.I., Lam P.K.S., Wang X.H., Yamashita N. (2020). Nationwide distribution and potential risk of bisphenol analogues in Indian waters. Ecotoxicol. Environ. Saf..

[131] Zhao X., Zhang H., Chen Z.L., Wang X.C., Shen J.M. (2020). Spatial and temporal distributions of bisphenol analogues in water and sediment from the Lanzhou section of the Yellow River, China. Arab. J. Geosci..

[132] Gao D.W., Wen Z.D. (2016). Phthalate esters in the environment: A critical review of their occurrence, biodegradation, and removal during wastewater treatment processes. Sci. Total Environ..

[133] Net S., Delmont A., Sempéré R., Paluselli A., Ouddane B. (2015). Reliable quantification of phthalates in environmental matrices (air, water, sludge, sediment and soil): A review. Sci. Total Environ..

[134] Wang Y.W., Jiang G.B., Lam P.K.S., Li A. (2007). Polybrominated diphenyl ether in the East Asian environment: A critical review. Environ. Int..

[135] Ohoro C.R., Adeniji A.O., Okoh A.I., Okoh O.O. (2021). Polybrominated diphenyl ethers in the environmental systems: A review. J. Environ. Health Sci. Eng..

[136] Soares A., Guieysse B., Jefferson B., Cartmell E., Lester J.N. (2008). Nonylphenol in the environment: A critical review on occurrence, fate, toxicity and treatment in wastewaters. Environ. Int..

[137] Bedoux G., Roig B., Thomas O., Dupont V., Le Bot B. (2012). Occurrence and toxicity of antimicrobial triclosan and by-products in the environment. Environ. Sci. Pollut. Res..

[138] Haman C., Dauchy X., Rosin C., Munoz J.-F. (2015). Occurrence, fate and behavior of parabens in aquatic environments: A review. Water Res..

[139] Kurwadkar S., Dane J., Kanel S.R., Nadagouda M.N., Cawdrey R.W., Ambade B., Struckhoff G.C., Wilkin R. (2022). Per- and polyfluoroalkyl substances in water and wastewater: A critical review of their global occurrence and distribution. Sci. Total Environ..

[140] Razzaque A.S., Melesse A.M. (2025). Global Insights into micro- and nanoplastic pollution in surface water: A review. Hydrology.

