# Supplementary material for: Research Progress on Genotoxicity and Molecular Mechanisms of Environmental Endocrine Disruptors
Source: Toxics. 2026 Sep 30;14(10):882. doi: 10.3390/toxics14100882 (PMC13709894; doi:10.3390/toxics14100882)
Supplement: Supplementary file 1 [file toxics-14-00882-s001.zip › toxics-4525692-supplementary.pdf]

Table S1
Summary of in vivo genotoxicity data of typical environmental endocrine disruptors (animal models).

Pollutants	Animal mode	Exposure dose	Exposure duration	Genotoxic endpoint	Experimental methods	Results	Ref
PCP	Red wiggler（*Eisenia fetida*）	15、30、45、60、75 mg/L	24、48、72 h	DNA Damage	Comet assay	Pentachlorophenol (PCP) exerts genotoxic effects at relatively low exposure concentrations ranging from 4.3 to 71.2 mg·L⁻¹; low-level PCP exposure can induce DNA damage. At non-lethal concentrations, significant impairment of multiple physiological functions and reduced activity are observed in Eisenia fetida.	Zhang et al., 2011
BPA	Blackfin goodea（*Goodea atripinnis*）	1、10、50、75 mg/L	14 d、28 d	DNA Damage	Comet assay	Exposure to sublethal concentrations of Bisphenol A (BPA) induces DNA damage in testicular cells (somatic cells and germ cells) and ovarian cells of Goodea atripinnis.	Cervantes-Camacho et al., 2020
CBZ	rare minnow（*Gobiocypris Rarus*）	1、10、100 μg/L	28 d	DNA Damage	Comet assay	Environmentally relevant concentrations of carbamazepine (CBZ) induce oxidative DNA damage in the liver of Gobiocypris rarus and trigger cellular apoptosis via the Ras/Raf/ERK/p53 signaling pathway.	Yan et al., 2021
MT	Nile tilapia（*Oreochromis niloticus*）	0.01、0.1、1.0 mg/L	96 h	DNA Damage、Chromosomal aberration	Comet assay Micronucleus assay	At the lowest exposure concentration of 0.01 mg/L, MT induced micronuclei in both test species, whereas no significant alterations were detected via the alkaline comet assay.	Rivero-Wendt et al., 2013
TMDD	Zebrafish（*Danio rerio*）	20、40、60、80、100 mg/L<br>40，80、160 mg/L	48 h	DNA Damage、Chromosomal aberration	Comet assay Micronucleus assay	TMDD induces DNA strand breaks at concentrations ≥ 100 mg/L, while no evidence of genotoxicity was observed within this concentration range via the cytokinesis-block micronucleus assay.	Vincze et al., 2014
E2	Nile tilapia（*Oreochromis niloticus*）	6 ng/L	24 h，48 h、10 d	DNA Damage、Chromosomal aberration	Comet assay Micronucleus assay	Acute and chronic exposure to 17β-estradiol exerts mutagenic and genotoxic effects on blood cells of Oreochromis spp.	Sponchiado et al., 2011
BPA	Wistar（*Rattus norvegicus*）	200 mg/(kg bw·d)	10 d	DNA Damage	Comet assay Meiotic chromosome spreading	Exposure to bisphenol A (BPA) may trigger the accumulation of DNA damage in rat germ cells via oxidative stress. Melatonin represents a promising pharmacological candidate for the prevention of potential genotoxicity induced by occupational or environmental BPA exposure.	Wu et al., 2013
ATZ	Japanese quail（*Coturnix japonica*）	0-500 mg / kg bw	15 d，30 d、45 d	DNA Damage	Comet assay	Hepatocytes and lymphocytes exhibited DNA damage. Significantly elevated comet tail length was observed in quails subjected to 45-day oral administration of atrazine (ATZ) at a dose of 500 mg/kg body weight (bw).	Hussain et al., 2011
BPA	BALB/c mouse（*Mus musculus*）	3 μM 、10 μM、30 μM、50 μM	24 h	DNA Damage、Chromosomal aberration	Comet assay Micronucleus assay	Bisphenol A (BPA) exhibits genotoxicity in murine macrophages in a concentration- and time-dependent manner.	Huang et al., 2018
BPA	Common carp（*Cyprinus carpio*）	3、4.5、6 mg / L	30 d	DNA Damage	Comet assay	Significant genotoxicity was detected in liver, brain, gill and kidney tissues of common carp. The observed genotoxicity (nuclear damage) in carp may be attributed to elevated generation of free radicals and oxidative stress, whereas the detailed underlying mechanisms at cellular and molecular levels remain unclear.	Afzal et al., 2022
Metformin	Rohu（*Labeo rohita*）	40 、80 μg/L	28 d	DNA Damage	Comet assay	Metformin triggers intracellular reactive oxygen species (ROS) overproduction, elicits oxidative stress, and subsequently causes DNA strand breaks and DNA damage in hepatocytes, with the severity of damage positively correlated with exposure concentration (high concentration: 80 μg/L > low concentration: 40 μg/L).	Sibiya et al., 2023
BPA	Water flea（*Daphnia magna*）	52、20 μM	24 h、48 h	DNA Damage	Comet assay	Bisphenol A (BPA) exerts significant cytotoxic and endocrine-disrupting effects on Daphnia magna cells by inducing oxidative stress, altering gene expression, and triggering DNA damage.	Sreevidya et al., 2025
ATR	Treefrog（*Dendropsophus minutus*）	0.02、0.04、0.08、2 μg/L	96 h	DNA Damage、Chromosomal aberration	Comet assay Micronucleus assay	Even at atrazine (ATZ) concentrations ≤ the Brazilian statutory limit of 2 μg/L, tadpoles of Dendropsophus minutus exhibited significant DNA damage and erythrocytic nuclear abnormalities (ENA), indicating that the current water quality criteria may be insufficient to protect amphibian health. Nevertheless, no obvious oxidative stress or metabolic disturbance was observed under 96 h acute exposure, suggesting that genotoxicity could serve as a more sensitive early warning biomarker.	Lopes et al., 2025
BPA	Common carp（*Cyprinus carpio*）	1.31 mg/L	6 w	DNA Damage	Comet assay	Chronic exposure to bisphenol A (BPA) can induce neurotoxicity in common carp, manifested as oxidative stress, immunosuppression, blood–brain barrier damage, acetylcholinesterase (AChE) inhibition, DNA damage and cellular apoptosis.	Tahir et al., 2024
BPs	Zebrafish（*Danio rerio*）	24h: 50, 100, 200 μM	24、96 h	DNA Damage	Comet assay	BPAF, BPAP and BPPH exhibited stronger cytotoxicity and genotoxicity than bisphenol A (BPA) in the ZFL 3D spheroid model.	Štern et al., 2025

		96h: 10, 25, 50, 100 μM					
DiPeP	Silver catfish （*Rhamdia quelen*）	5、25、125 ng/g	30 d	DNA Damage	Comet assay	Significant DNA damage was detected via the alkaline comet assay in the head kidney across all exposure dose groups, whereas no notable genotoxic lesions were observed in other tissues including blood, liver, trunk kidney and brain.	Oya-Silva et al., 2023

Afzal, G.; Ahmad, H.I.; Hussain, R.; Jamal, A.; Kiran, S.; Hussain, T.; Saeed, S.; Nisa, M.u. Bisphenol A Induces Histopathological, Hematobiochemical Alterations, Oxidative Stress, and Genotoxicity in Common Carp (*Cyprinus carpio* L.). *Oxid. Med. Cell. Longev.* 2022, 2022, 5450421.

Cervantes-Camacho, I.; Guerrero-Estévez, S.M.; López, M.F.; Alarcón-Hernández, E.; López-López, E. Effects of Bisphenol A on Foxl2 gene expression and DNA damage in adult viviparous fish *Goodea atripinnis*. *J. Toxicol. Environ. Health A* 2020, 83, 95–112.

Huang, F.M.; Chang, Y.C.; Lee, S.S.; Ho, Y.C.; Yang, M.L.; Lin, H.W.; Kuan, Y.H. Bisphenol A exhibits cytotoxic or genotoxic potential via oxidative stress-associated mitochondrial apoptotic pathway in murine macrophages. *Food Chem. Toxicol.* 2018, 122, 215–224.

Hussain, R.; Mahmood, F.; Khan, M.Z.; Khan, A.; Muhammad, F. Pathological and genotoxic effects of atrazine in male Japanese quail (*Coturnix japonica*). *Ecotoxicology* 2011, 20, 1–8.

Lopes, A.T.C.; Benvindo-Souza, M.; Sotero, D.F.; Pedroso, T.M.A.; Tomaz, A.A.; Costa, A.G.; Gois, A.R.S.; Vieira, T.B.; Bastos, R.P.; Silva, D.M.E. Sublethal effects of atrazine concentrations exposure on tadpoles of *Dendropsophus minutus*: Evaluation of redox status, micronuclei frequencies and comet assay as biomarkers. *Aquat. Toxicol.* 2025, 279, 107260.

Oya-Silva, L.F.; Guiloski, I.C.; Vicari, T.; Deda, B.; Marcondes, F.R.; Simeoni, R.D.; Perussolo, M.C.; Martino-Andrade, A.J.; Leme, D.M.; de Assis, H.C.S.; Cestari, M.M. Evidence of genotoxicity, neurotoxicity, and antioxidant imbalance in silver catfish *Rhamdia quelen* after subchronic exposure to diisopentyl phthalate. *Mutat. Res.-Genet. Toxicol. Environ. Mutagen.* 2023, 892, 503702.

Rivero-Wendt, C.L.G.; Miranda-Vilela, A.L.; Ferreira, M.F.N.; Amorim, F.S.; Silva, V.A.G.; Louvandini, H.; Grisolia, C.K. Lack of genotoxicity in *Astyanax bimaculatus* and *Oreochromis niloticus* of 17α-methyltestosterone used in fish hatcheries to produce male monosex populations. *Genet. Mol. Res.* 2013, 12(4), 5013–5022.

Sibiya, A.; Al-Ghanim, K.A.; Govindarajan, M.; Nicoletti, M.; Sachivkina, N.; Vaseeharan, B. Biochemical patterns and genotoxicity of the endocrine disruptor metformin in the freshwater fish *Labeo rohita*. *Fishes* 2023, 8, 380.

Sponchiado, G.; Reynaldo, E.M.F.L.; Andrade, A.C.B.; Vasconcelos, E.C.; Adam, M.L.; Oliveira, C.M.R. Genotoxic effects in erythrocytes of *Oreochromis niloticus* exposed to nanograms-per-liter concentration of 17β-estradiol (E_2): An assessment using micronucleus test and comet assay. *Water, Air, Soil Pollut.* 2011, 218, 353–360.

Sreevidya,C.P.; Manoj, K.T.M.; Balakrishnan, S.; Kunjiraman,S.; Sarasan, M.; Magnuson, J.T.; Puthumana, J. Establishment of a cell culture from *Daphnia magna* as an in vitro model for (eco)toxicology assays: Case study using Bisphenol A as a representative cytotoxic and endocrine disrupting chemical. *Aquat. Toxicol.* 2025, 278, 107173.

Štern, A.; Kološa, K.; Rozman, Š.; Žegura, B. Exploring the genotoxic potential of Bisphenol A and its emerging alternatives in an advanced in vitro 3D zebrafish hepatic cell model. *Sci. Total Environ.* 2025, 1001, 180527.

Tahir, R.; Samra; Afzal, F.; Khan, H.; Ghaffar, A.; Qazi, I.H.; Al-Khattaf, F.S.; Liulan, Z.; Yan, H.X.; Kuo, H.; Shrestha, A.; Jamil, H.; Naseer, S.; Habib, H.; Yang, S. Chronic bisphenol A induced neurotoxicity: Exposure risk, molecular fate within carp and its potential phytoremediation. *Sci. Total Environ.* 2024, 954, 175876.

Vincze, K.; Gehring, M.; Braunbeck, T. (Eco)toxicological effects of 2,4,7,9-tetramethyl-5-decyne-4,7-diol (TMDD) in zebrafish (*Danio rerio*) and permanent fish cell cultures. *Environ. Sci. Pollut. Res.* 2014, 21, 8233–8241.

Wu, H.J.; Liu, C.; Duan, W.X.; Xu, S.C.; He, M.D.; Chen, C.H.; Wang, Y.; Zhou, Z.; Yu, Z.P.; Zhang, L.; Chen, Y. Melatonin ameliorates bisphenol A-induced DNA damage in the germ cells of adult male rats. Mutat. Res.-Genet. Toxicol. Environ. Mutagen. 2013, 752, 57–67.

Yan, S.H.; Chen, R.; Wang, M.; Zha, J.M. Carbamazepine at environmentally relevant concentrations caused DNA damage and apoptosis in the liver of Chinese rare minnows (*Gobiocypris rarus*) by the Ras/Raf/ERK/p53 signaling pathway. *Environ. Pollut.* 2021, 270, 116245.

Zhang, R.Y.; Yang, J.; Liu, X. Ecotoxic effects and genotoxicity of pentachlorophenol. *Chin. Environ. Chem.* 2011, 30, 476–481. (in Chinese)

Table S2

Summary of in vitro genotoxicity data of typical environmental endocrine disruptors (human cell lines).

Pollutants	Animal mode	Exposure dose	Exposure duration	Genotoxic endpoint	Experimental methods	Results	Ref
DCBQ 、DCMBQ 、TCBQ、DBBQ	5637 human bladder cancer cells；Caco-2 human colon adenocarcinoma cells；MGC-803 human gastric carcinoma cells	25–150 μM	24 h	Chromosomal aberration	Micronucleus assay	The lowest effective concentrations for micronucleus induction by DCBQ, DCMBQ, TCBQ and DBBQ in cell lines were 50–75, 20–41.5, 87.4–100 and 50 μM, respectively. HBQ is capable of triggering chromosomal damage. Among all tested compounds, DCMBQ exerted the strongest cytotoxicity and genotoxicity across all cell lines, while TCBQ exhibited the weakest toxic effects.	Li et al., 2020
FA、ACR	A549 cell	FA：80、100 120、140 μM ACR：55、65、75、85 μM	24 h	DNA Damage、Chromosomal aberration	Comet assay、Micronucleus assay	Significant combined cytotoxic and genotoxic effects were observed for the mixture of FA and ACR in A549 cells at subcytotoxic concentrations, and prolonged exposure poses severe threats to human health.	Zhang et al., 2018
FA、ACR	BEAS-2B human Bronchial epithelial cells	FA：5、10、20、40、80 μM ACR：0.5、1、2、4、6、7.5 μM	1 h、12 h、24 h	DNA Damage、Chromosomal aberration	Comet assay、Micronucleus Assay、γH2AX assay	Even at smoking-relevant concentrations, aldehyde mixtures induce multiple combined genotoxic effects in BEAS-2B cells, which vary depending on the genotoxic endpoint assessed. Such mixtures may pose more severe risks to the respiratory system of animals and humans than would be predicted based on the toxicity of individual aldehydes.	Zhang et al., 2020
PBS	peripheral blood mononuclear cells(PBMCs)	200 mg/mL	48 h、72 h	Chromosomal aberration	Micronucleus assay	Chromosomal aberration tests with 200 mg/mL PBS yielded negative results, and the number of micronucleated cells induced showed no statistically significant differences. Therefore, under the experimental conditions of this study, the extract of biodegradable PBS material exhibited no genotoxicity toward peripheral blood mononuclear cells (PBMCs) in vitro, with effects comparable to medical-grade L-PLA.	Duan et al., 2014
Prochloraz	Caco-2 human colon adenocarcinoma cells	5、50、75、150、300 μM	24 h、48 h	DNA Damage、Chromosomal aberration	Comet assay、Micronucleus Assay、γH2AX assay	Three distinct test systems have verified that acetamiprid possesses cytotoxic and genotoxic potential in human intestinal cells, yet the exact genotoxic mechanisms remain unclear.	Çinkılıç et al., 2012
DINCH	HepG2 HK-2	1、10、100、250、500 μM	6 h、48 h	DNA Damage、Chromosomal aberration	Comet assay、Micronucleus assay	DINCH induces renal cytotoxicity and transient oxidative DNA damage in hepatocytes.	Pérez-Albaladejo et al., 2019
Prochloraz	H295R HepG2	1、10、50、100、150 μM	4 h、24 h	DNA Damage	Comet assay	Prochloraz at non-cytotoxic concentrations is capable of inducing oxidative stress and DNA damage in cultured human cells, indicating that prochloraz may be genotoxic.	Lundqvist et al., 2016
PFAS	HepG2	0.93 μM–62.1 μM	24 h	DNA Damage	Comet assay	The DNA damage induced by individual PFAS compounds and PFAS mixtures in the present study was mild to moderate, which was substantially lower than the damage level induced by the positive control (10 μM H_2O_2); additionally, no clear dose-dependent relationship was observed for such damage.	Ojo et al., 2022
BPs	HepG2	BPAF：1.25、2.5、5、10 μM BPA：20 μM BPF：25、50 μM BPP：25 μM	72 h	DNA Damage	Comet assay	BPAF significantly induced DNA strand breaks at 1.25 μM, exhibiting the strongest genotoxicity. BPA (20 μM), BPF (25 and 50 μM), and BPP (25 μM) markedly elevated the Olive tail moment (OTM), resulting in evident DNA damage.	Wang et al., 2025
BDE-99	HepG2	5~40 μM	48 h	Chromosomal aberration	Micronucleus assay	Sole exposure to BDE-99 produced no cytotoxicity and failed to induce micronuclei. Following pretreatment with either BPA or CITCO, BDE-99 induced micronuclei in a concentration-dependent manner, and all observed micronuclei lacked CENP-B expression, indicating clastogenic damage stemming from chromosome breakage rather than chromosome loss. At the highest tested concentration (40 μM), the frequency of CENP-B-negative	Wang et al., 2024

						micronuclei increased significantly (P < 0.01).	
DMP、DEP、DBP、BBP、DEHP、BPA、NP	HepG2	1、10、100、1000 μg/L	4 h、24 h	DNA Damage	Comet assay	After 4 h exposure, significant DNA damage was induced by BBP and DEHP at concentrations ≥ 1 μg/L, by DMP, DEP, DBP and BPA at concentrations ≥ 10 μg/L, and by NP at concentrations ≥ 100 μg/L. After 24 h exposure, DBP, BPA and NP triggered remarkable DNA damage starting at 10 μg/L, while DMP, DEP, BBP and DEHP induced evident DNA damage at concentrations ≥ 100 μg/L. All compounds exerted genotoxic effects in a dose-dependent manner.	Balabanič et al., 2021

Balabanič, D.; Filipič, M.; Krivograd Klemenčič, A.; Žegura, B. Genotoxic activity of endocrine disrupting compounds commonly present in paper mill effluents. *Sci. Total Environ.* 2021, 794, 148489.

Çinkılıç, Ç.; Velioglu, T.; Çiçek, N.; Cavas, T. In vitro genotoxicity evaluation of acetamiprid in CaCo-2 cells using the micronucleus, comet and γH2AX foci assays. *Pestic. Biochem. Physiol.* 2012, 104, 212–217.

Duan, C.E.; Gong, B.; Yang, X.B. In vitro genotoxicity of poly(butylene succinate) extract toward human peripheral blood mononuclear cells. *Chin. J. Tissue Eng. Res.* 2014, 18, 5475–5480. (in Chinese)

Li, Y.B.; Zhang, H.F.;Han, Y.N.; Chao, H.; Ma, M.;Yang, M. Cytotoxicity and genotoxicity assays of halobenzoquinones disinfection byproducts using different human cell lines. *Chemosphere* 2020, 256, 127061.

Lundqvist, J.; Hellman, B.; Oskarsson, A. Fungicide prochloraz induces oxidative stress and DNA damage in vitro. *Food Chem. Toxicol.* 2016, 91, 36–41.

Ojo, A.F.; Peng, C.; Ng, J.C. Genotoxicity assessment of per- and polyfluoroalkyl substances mixtures in human liver cells (HepG2). *Toxicology* 2022, 482, 153359.

Pérez-Albaladejo, M.; Porte, C.; Fernandes, M.S. In vitro exposure to the next-generation plasticizer diisononyl cyclohexane-1,2-dicarboxylate (DINCH): cytotoxicity and genotoxicity assessment in human cells. *J. Toxicol. Environ. Health A* 2019, 82, 817–831.

Wang, J.; Hong, X.S.; Liu, W.; Zhang, L.; Yan, S.H.; Li, Z.T.; Zha, J.M. Comprehensive assessment of the safety of bisphenol A and its analogs based on multi-toxicity tests in vitro. *J. Hazard. Mater.* 2025, 486, 136983.

Wang, L.; Murtala, N.M.; Hu, K.Q.; Chen, Y.J.; Chen, M.X.; Sun, H.T.; Liu, Y.G. Prediction of polybrominated diphenyl ethers (PBDEs) as potential substrates of various human CYP enzymes and laboratory test of BDE-99 for its metabolism-activated mutagenicity. *Toxicology* 2024, 509, 153992.

Zhang, S.; Chen, H.; Wang, A.; Liu, Y.; Hou, H.W.; Hu, Q.Y. Combined effects of co-exposure to formaldehyde and acrolein mixtures on cytotoxicity and genotoxicity in vitro. *Environ. Sci. Pollut. Res.* 2018, 25, 25306–25314.

Zhang, S.; Chen, H.; Zhang, J.N.; Liu, J.; Hou, H.W.; Hu, Q.Y. The multiplex interactions and molecular mechanism on genotoxicity induced by formaldehyde and acrolein mixtures on human bronchial epithelial BEAS-2B cells. *Environ. Int.* 2020, 144, 107401.
